# Feasibility of Digital Augmentation of Parent-Child Interaction Therapy: A Randomized Clinical Trial

**DOI:** 10.1001/jamanetworkopen.2025.48869

**Published:** 2025-12-15

**Authors:** Magdalena Romanowicz, Maria T. Saliba, Angelina R. Wilton, Juan F. Garzon Hincapie, Kyle S. Croarkin, Christina T. Saliba, Allison LeMahieu, Noelle Drapeau, Brandi Schlichting, Michelle Skime, William V. Bobo, Jennifer L. Vande Voort, Julia Shekunov, Paul E. Croarkin, Arjun P. Athreya

**Affiliations:** 1 Department of Psychiatry and Psychology, Mayo Clinic, Rochester, Minnesota; 2 Department of Molecular Pharmacology and Experimental Therapeutics, Mayo Clinic, Rochester, Minnesota; 3 Center for Individualized Medicine, Mayo Clinic, Rochester, Minnesota; 4 Department of Quantitative Health Sciences, Mayo Clinic, Rochester, Minnesota; 5 Department of Behavioral Sciences and Social Medicine, Florida State University, Tallahassee; 6 Mayo Clinic Children’s, Mayo Clinic, Rochester, Minnesota

## Abstract

**Importance:**

The potential utility of real-time digital therapeutics that identify impending behavioral outbursts to proactively apply evidence-based interventions for managing children with disruptive behavior disorder has not been tested.

**Objectives:**

To evaluate feasibility and adherence of a digitally augmented, real-time, proactive behavioral intervention (smartwatch) for children with disruptive behaviors.

**Design, Setting, and Participants:**

This randomized clinical trial was conducted between March 1, 2022, and December 31, 2023, at Mayo Clinic in Rochester, Minnesota. The study enrolled children aged 3 to 7 years with externalizing behavior problems rated above the clinically significant range (I score ≥120; T score ≥60) on the Eyberg Child Behavior Inventory (ECBI).

**Interventions:**

Twelve weekly sessions of parent-child interaction therapy (PCIT) (treatment as usual [TAU] arm) vs artificial intelligence (AI)–enhanced PCIT augmented with real-time behavioral outburst alerts (PCIT-AI arm).

**Main Outcomes and Measures:**

The primary outcome was achieving the a priori feasibility benchmark set at greater than 70% for both completion of PCIT sessions and daily smartwatch wear. Parental reaction time to behavior prompts was measured in seconds. Secondary outcomes were percentage changes in ECBI and Pediatric Sleep Questionnaire scores. Behavioral outburst duration was examined based on parental logs of start and end times of observed tantrums using a smartphone application.

**Result:**

This study enrolled 50 children (median [IQR] age, 5.0 [4.0-6.0] years; 34 [68%] male and 16 [32%] female; 28 randomized to the PCIT-AI arm and 22 to the PCIT-TAU arm). Of these children, 37 (74%; 21 in the PCIT-AI arm and 16 in the PCIT-TAU arm) completed PCIT, with children wearing the watch a median (IQR) 75.7% (67.8%-82.9%) of the time, thus achieving the feasibility benchmark (primary outcome). Median (IQR) response time to behavior prompts in the PCIT-AI arm was 3.65 (2.07-7.08) seconds. Assessing between-group differences, there was no statistically significantly greater numerical improvement in ECBI-intensity percentage change (13.86%; 95% CI, −1.41% to 29.14%), ECBI-problem percentage change subscale scores (38.59%; 95% CI, −1.22% to 78.40%), or absolute change in Pediatric Sleep Questionnaire scores (−0.16; 95% CI, −1.85 to 1.53) with the PCIT-AI arm than with the PCIT-TAU arm. Children in the PCIT-AI arm had significantly shorter mean (SD) tantrum durations (10.4 [20.8] minutes vs 22.1 [30.0] minutes; *P* < .001) and lower odds of tantrums lasting for 15 minutes or more (odds ratio, 3.66; 95% CI, 2.72-4.95).

**Conclusions and Relevance:**

In this randomized clinical trial, both recruitment and technology feasibility benchmarks were achieved. The findings inform the design of fully powered efficacy study of wearable, digitally augmented PCIT for children with disruptive behaviors.

**Trial Registration:**

ClinicalTrials.gov Identifier: NCT05077722

## Key Points

**Question:**

Can parents and children use real-time digital therapeutic augmentation of behavior therapy via smartwatch for proactively applying evidence-based parenting skills when temper tantrums are anticipated to occur?

**Findings:**

This randomized clinical trial of 50 children with externalizing behavior problems achieved recruitment benchmark and demonstrated that delivering digital intervention was feasible. In families completing parent-child interaction therapy, children wearing the smartwatch exceeded the adherence benchmark (primary outcome), and parents responded to behavior prompts for proactive parenting skills in less than 4 seconds.

**Meaning:**

The findings inform the design of fully powered future efficacy study of wearable-based digitally augmented parent-child interaction therapy.

## Teaser

This randomized clinical trial evaluates the feasibility and adherence of a digital therapeutic augmentation of behavior therapy for children with externalizing behavior problems.

## Introduction

Disruptive behaviors in young children are characterized by emotion dysregulation, defiant behaviors, aggression, and intense emotional outbursts (referred to as tantrums in this work) that exceed normative patterns with regard to their intensity, frequency, and/or duration.^1,2,3^ Disruptive behaviors are expressed across several common disorders impacting children, including oppositional defiant disorder, attention-deficit/hyperactivity disorder (ADHD), autism spectrum disorder (ASD), and mood disorders. Affected children require significantly more time and effort to recover from tantrums than unaffected age-matched peers.^4^ In preschoolers, tantrums rarely last longer than 15 minutes,^5^ whereas those lasting longer than 25 minutes are more characteristic of serious mental illnesses.^6^ Parenting interventions imparted in contemporary evidence-based child behavior therapies (eg, parent-child interaction therapy [PCIT]^7,8,9,10,11^) have the potential to successfully mitigate tantrums and signs of behavioral dyscontrol. However, such steps are typically reactive (ie, implemented after tantrums have begun) and are contingent upon a parent’s ability to rapidly recall and effectively use PCIT skills under emotionally intense conditions, factors that may limit intervention effectiveness.^12,13^ Digital therapeutics (DTs) have the potential to improve this situation by providing early signals of impending tantrums using physiologic data from sensors embedded in wearable devices. In so doing, PCIT interventions may be applied proactively and with greater effectiveness before tantrums fully unfold. The success of this approach is predicated on proactive parental implementation of PCIT practices and adequate patient adherence to DT-enabling wearable devices.

Despite the wide availability of commercially available wearables and mobile apps for mental health, digital health–based interventions for pediatric mental health are limited,^14^ and the efficacy of such approaches are often limited by human factors, especially low adherence to wearables.^15,16,17^ Therefore, effective trials of DTs for disruptive behavior in children should address device adherence as a primary study end point in addition to ensuring the enrollment of naturalistic patient samples (ie, with comorbid conditions such as ADHD and ASD) and statistically accounting for factors that may limit treatment effects, including patient age and sex, concomitant medications, and parental educational, employment, and marital status.^18,19,20^

With these considerations in mind, this preregistered, parallel-group randomized clinical trial assessed feasibility of a wearable-based digital augmentation of PCIT to evaluate adherence during therapy and clinical effects for young children with disruptive behaviors. All participants received 12 sessions of PCIT, either with artificial intelligence (AI)–enhanced, real-time digital alerts for impending tantrums (PCIT-AI) or without digital alerts (PCIT-TAU [treatment as usual]).^21^ The study assessed the feasibility benchmark of adherence (percentage of smartwatch wear time) as the primary outcome and behavioral (Eyberg Child Behavior Inventory [ECBI]^22^) and sleep (Pediatric Sleep Questionnaire [PSQ]) measures as secondary outcomes. Parent-reported tantrum durations were also recorded using a mobile app and were compared between the study arms. To facilitate proactive PCIT implementation, parents in the PCIT-AI arm had access to a mobile app that generated smartphone cues for attending to their child based on previously published work estimating increased tantrum risk from specific time window variations in heart rate.^23^ The study hypothesized that at least 70% of enrolled children would achieve a smartwatch wear time of 70% or more of the treatment period (the threshold used to define adequate adherence^24,25,26^).

## Methods

### Study Design and Participants

This was a therapist-, patient-, and parent- and/or caregiver- blinded, parallel-group randomized clinical trial conducted at Mayo Clinic between March 1, 2022, and December 31, 2023. The study was approved by the Mayo Clinic institutional review board. Written informed consent was obtained from parents or caregivers of each participant before randomization. Children aged 3 to 6 years provided oral assent, and children aged 7 years provided informed written assent. Consolidated Standards of Reporting Trials (CONSORT)^27^ guidelines were followed in this study report. The trial protocol, including details of the study procedures and retention strategies, can be found in Supplement 1 and has been published elsewhere.^21^

### Inclusion and Exclusion Criteria for Children and Parents

Children (aged 3-7 years) with clinically significant externalizing behavior problems (EBPs) (I score ≥120; T score ≥60) who were able to understand written or spoken English and were willing to wear a study smartwatch were enrolled. Children in foster care or those diagnosed with severe intellectual disability, ASD with accompanying severe language impairment, or any psychotic disorder were ineligible. Parents had to be willing to wear a study smartwatch and to understand written and spoken English. Those who were unwilling or unable to provide written consent and adhere to study procedures were deemed ineligible.

### Randomization and Blinding

Simple randomization was conducted (P.E.C.) using a random-number generator (50 consecutive numbers assigned) to allocate study participants into their respective treatment groups. Study participants, their parents, and the PCIT clinician (M.R.), who also performed clinical ratings, were blinded to the group assignment. Although all parents installed the same mobile app (Ilumivu’s mEMA app) for the study, a cloud-based management portal of the mobile app (only accessible to study team) ensured that only parents in the PCIT-AI arm received parental prompts. Parents in the PCIT-TAU group received only default messages on general well-being (ie, to stand or move often during day hours). Assessment of the blinding process was conducted using the James Blinding Index by asking both the therapist and parent to guess each child’s allocated treatment group.^28^

### Intervention

All participants received 12 sessions of PCIT (1 session per week) as part of routine care.^29^ PCIT is an evidence-based, family-centered intervention for children with disruptive behaviors delivered in 2 sequential phases: child-directed intervention, in which parents practice positive interactions and ways to ignore minor misbehaviors, and parent directed intervention, in which parents learn safe discipline strategies, including timeouts. Children were allowed to start and stop medications during the study to reflect naturalistic treatment and bolster the generalizability of the study. A PCIT clinician (M.R., a board-certified child and adolescent psychiatrist) managed all psychotropic medications during the study. Children with nonsevere ASD could receive applied behavioral analysis therapy concurrently with PCIT as a standard-of-care intervention. Concomitant play therapy, child parent psychotherapy, and related therapies had to be paused or discontinued prior to starting PCIT in this trial.

### Outcome Measures

The primary study outcome demonstrating feasibility of delivering digital intervention was adherence. Adherence was defined as the percentage of time that the study smartwatch was worn per day by participants during PCIT (automatically calculated by the smartwatch based on detecting a pulse). The expected adherence was 70% or more across the duration of PCIT. This threshold was based on the level of adherence needed in continuous monitoring of physiologic and biological measures with wearables in other chronic conditions for meaningful imputation.^24,25,26^ Response time was defined as the elapsed time (in seconds) from alert generation to parental response (opening the notification). Secondary outcomes were the percentage of changes on the ECBI intensity and problem subscales (comparing scores at enrollment and 1 week after the 12th PCIT session) and absolute changes in PSQ scores between the same time points. Both ECBI and PSQ were completed by the participating parent prior to the start of each PCIT session. The intensity subscale of ECBI captures frequency of the child’s behaviors, and problem subscale captures whether the identified behavior is a problem.^22^ Tantrum durations (minutes) were also compared between the PCIT-AI and PCIT-TAU arms based on parental reports recorded using an Ilumivu mobile app (eAppendix 1 in Supplement 1). A detailed list of assessments in the study is provided elsewhere.^21^

### Digital Health Setup and Adverse Reactions

All participants were issued a vivosmart 4 smartwatch (Garmin Ltd), the data from which were collected and stored via a central database (Fitabase). The study team verified a comfortable (sufficient gap for skin to be breathable and heart rate sensor to function) and snug fit of the smartwatch on the child participant’s arm. Participants’ smartwatches were paired to the parents’ smartphones (or a study-provided smartphone if the parents did not possess one), which permitted the use of continuous heart rate data from the participant’s smartwatch to trigger a warning on the parent’s smartphone (via an Ilumivu app) of an impending tantrum (if the child was in the PCIT-AI arm). A message was triggered on the parent’s smartphone if the child’s smartwatch sensed an mean heart rate (within a 10-minute window) of 105 beats per minute to 129 beats per minute between 7 am and 8 pm (PCIT-AI arm only). The range of heart rate to trigger a parental action was based on prior work that established the ability of smartwatch-derived biomarkers to classify children’s behaviors.^23^ Parents were prompted to direct attention to their child and initiate PCIT-directed interventions upon receiving a notification from the smartphone app regarding an impending tantrum (PCIT-AI arm only). Three reminders to check the real-time alerts were sent at 20-minute intervals. See eAppendix 2 in Supplement 1 for additional information on technology support provided during the study. Participants were requested to report any harms or adverse effects from technology use.

### Sample Size

The analytic plan for this study was preregistered and published elsewhere.^21^ A wear time of 70% or more during the 12 weeks of PCIT was the prespecified definition of adequate adherence for the primary study aims and hypothesis. For the secondary aim (clinical outcome with ECBI), it was determined that 25 participants per group would provide 70% power, with α = .05 alpha (2-tailed) to detect behavior changes and an odds ratio (OR) of 2.0 favoring PCIT-AI. This relatively low threshold for statistical power was acceptable in the context of a study focused on practicality as opposed to definitive testing of clinical efficacy.

### Statistical Analysis

Demographic and clinical characteristics are summarized using descriptive statistics, overall and by treatment arm. We used 2-tailed, unpaired *t* tests or Wilcoxon rank sum tests (for continuous variables) and χ^2^ or Fisher exact test (for categorical variables) for comparisons between the treatment arms. With the primary goal of assessing feasibility, utilization, and efficacy of the digital health technology ecosystem, the intention-to-treat samples included participants who were randomized to either intervention arm and completed at least 1 PCIT session.

Percentage of wear time (adherence) and nights slept with the smartwatch were analyzed using 2-sample *t* tests. Continuous outcomes (ECBI changes and tantrum durations in minutes) were analyzed using generalized linear models. Analyses were adjusted to account for potential confounding by baseline value, child’s age and sex,^18^ and comorbid ADHD. Cox proportional hazards regression models were used to estimate study completion between the randomized treatment groups. A Kaplan-Meier plot was used to visualize the dropout rates during the study by treatment group. James Blinding Indexes for both the PCIT clinician and parents was obtained to assess whether satisfactory blinding was achieved, with possible scores ranging from 0 (unblinded) to 1 (perfect blinding). Satisfactory blinding was defined based on a 95% CI lower bound value greater than 0.5 for blinding index scores.^28^ Differences in tantrum durations between the treatment arms were analyzed using a mixed-effects model wherein child’s age, sex, and comorbid ADHD were considered as fixed effects and the child and week into therapy were nested as random effects. ORs (with 95% CIs) for tantrums lasting 15 minutes or more or 25 minutes or more were computed using the Fisher exact tests. Cohen *d* was used to estimate the effect sizes of ECBI changes between the randomized treatment arms. In all cases, a 2-sided *P* ≤ .05 was considered statistically significant, with no adjustment for multiple comparisons given the pilot nature of this study. Complete case analysis was used because the outcomes were measured on completing PCIT. Statistical analyses were performed using SAS software, version 9.4 (SAS Institute Inc) and R, version 4.1.2 (R Project for Statistical Computing).

## Results

### Enrollment, Participant Characteristics, and Adverse Reactions

**Figure 1.**
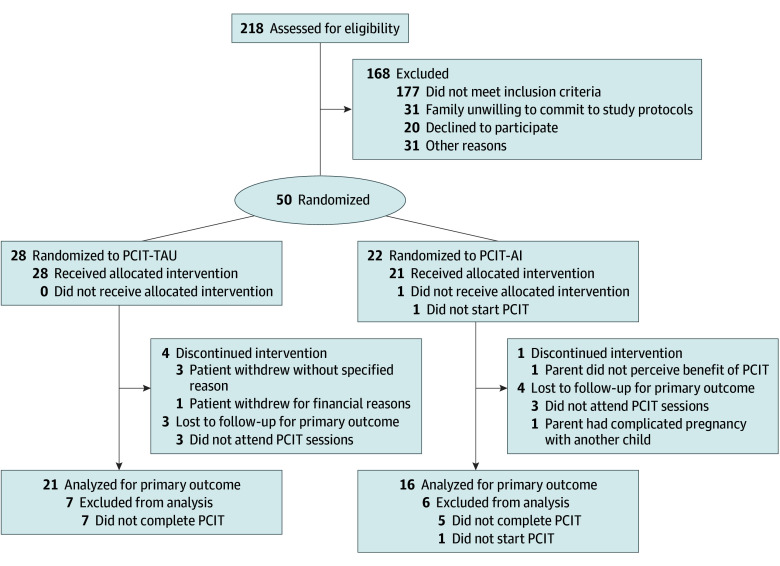
Flow Diagram of the PISTACHIO (Preemption of Disruptive Behavior in Children) Study PCIT indicates parent-child interaction therapy; PCIT-AI, artificial intelligence–enhanced parent-child interaction therapy; PCIT-TAU, treatment as usual parent-child interaction therapy.

A total of 218 children were screened for eligibility and 50 were randomized (median [IQR] age, 5.0 [4.0-6.0] years; 34 [68%] male and 16 [32%] female), including 28 in the PCIT-AI arm and 22 in the PCIT-TAU arm (Figure 1). The characteristics of study participants are summarized in the Table. ADHD was the most common condition present in the enrolled children (27 [54%]), and 25 participants (50%) were treated with stimulants. Five children were diagnosed with ASD (all in the PCIT-AI arm).

**Table.** Demographic Characteristics of the Study Participants

Characteristic	No. (%) of participants^a^		
	Overall (N = 50)	PCIT-AI (n = 28)	PCIT-TAU (n = 22)
Child age, median (IQR), y	5.0 (4.0-6.0)	5.0 (4.0-5.5)	4.5 (4.0-6.0)
Child sex			
Male	34 (68)	18 (64)	16 (73)
Female	16 (32)	10 (36)	6 (27)
Child diagnoses			
ADHD	27 (54)	15 (54)	12 (55)
ASD	5 (10)	5 (18)	0 (0)
Anxiety	7 (14)	3 (11)	4 (18)
SAD	5 (10)	2 (7)	3 (14)
Child medications			
Antidepressants	4 (8)	0 (0)	4 (18)
Stimulants	25 (50)	13 (46)	12 (55)
Nonstimulants	21 (42)	12 (43)	9 (41)
Parent age, median (IQR), y	35.0 (32.0-38.0)	35.0 (30.0-38.0)	36.0 (32.0-40.0)
Parent sex			
Male	43 (86)	24 (86)	19 (86)
Female	7 (14)	4 (14)	3 (14)
Marital status			
Single	2 (4)	2 (7)	0 (0)
Married	35 (70)	20 (71)	15 (68)
Separated or divorced	12 (24)	6 (21)	6 (27)
Widowed	1 (2)	0 (0)	1 (5)
Educational status			
High school degree	8 (16)	4 (14)	4 (18)
Some post high school training or some college	15 (30)	6 (21)	9 (41)
4-y College degree	11 (22)	4 (14)	7 (32)
Beyond 4-y college degree	16 (32)	14 (50)	2 (9)
Both parents in study	27 (54)	13 (46)	14 (64)
Baseline ECBI intensity subscale score, median (IQR) (n = 49)	161.0 (144.0-179.0)	161.5 (144.0-176.5)	158.0 (136.0-186.0)
Baseline ECBI problem subscale score, median (IQR) (n = 49)	19.0 (13.0-26.0)	18.5 (12.0-25.0)	19.0 (15.0-26.0)

Abbreviations: ADHD, attention-deficit/hyperactivity disorder; ASD, autism spectrum disorder; ECBI, Eyberg Child Behavior Inventory; PCIT-AI, artificial intelligence–enhanced parent-child interaction therapy; PCIT-TAU, treatment as usual parent-child interaction therapy; SAD, seasonal affective disorder.

^a^ Unless otherwise indicated.

Twenty-one children (75%) in the PCIT-AI group and 16 (73%) in the PCIT-TAU group completed all 12 sessions of PCIT. There were no significant between-group differences in study completion rates (hazard ratio, 0.87; 95% CI, 0.29-2.59) (eFigure 1 in Supplement 2). Among the 37 completers, 19 (51%) completed the 12 PCIT sessions in 16 weeks (eFigure 2 in Supplement 2). The James Blinding Indexes were 0.68 (95% CI, 0.55-0.81) in PCIT clinicians and 0.76 (95% CI, 0.63-0.89) in parents, indicating satisfactory blinding given that the lower bounds of the 95% CIs exceeded 0.5.

Only one participant characteristic was associated with study dropout. If both parents participated in the study, odds of a family quitting the study were 0.16 times that if only one parent was involved in the study (95% CI, 0.04-0.70; *P* = .02). With 2 participants each in both arms of the study not starting therapy, there were 46 participants in the samples for the intention-to-treat analyses for primary and secondary outcomes.

### Primary Outcome: Smartwatch Adherence as a Feasibility Benchmark

Among the 37 participants (74%) who completed the study, the median (IQR) smartwatch wear time was 75.7% (67.8%-82.9%) for the entire sample (79.6% [71.4%-91.1%] in the PCIT-AI arm vs 71.5% [62.5%-78.7%] in the PCIT-TAU arm; *P* = .01). For the entire sample and each arm individually, the median (IQR) nights per week that the watch was worn during sleep was 7 (7.0-7.0; *P* = .46).

Among the intention-to-treat samples, the median (IQR) smartwatch wear time was 74.6% (66.5% 80.3%) for the entire sample (6.9% [69.4%-89.0%] for the PCIT-AI arm vs 70.1%, [60.1%-78.0%] for the PCIT-TAU arm, *P* = .01). The adherence level in children who did not complete the study owing to unavailability for follow-up or incomplete PCIT participation (children not starting PCIT or parents not attending PCIT sessions) was 39.6%. No serious adverse reactions were reported in the study, and technology-related reports (eg, battery retention) from families are described in eAppendix 3 in Supplement 2.

### Secondary and Exploratory Outcomes: Child Behavior, Sleep, and Tantrum Duration

**Figure 2.**
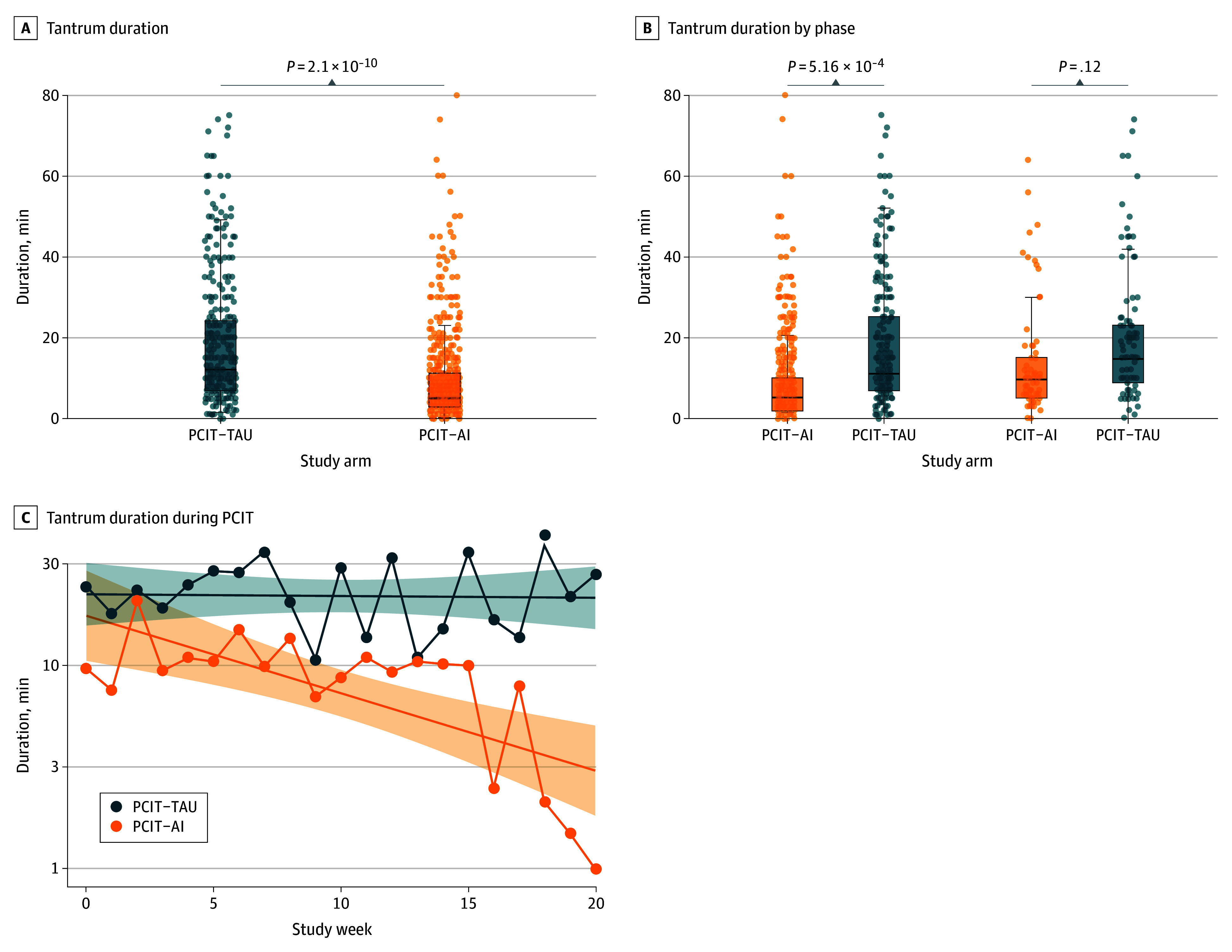
Difference in Duration of Tantrums Between Treatment Arms and Stratified by Child-Directed Intervention (CDI) and Parent-Directed Intervention (PDI) Phases of Parent-Child Interaction Therapy (PCIT) A and B, The ends of the boxes represent the 25th and 75th percentiles. The horizontal line inside the box indicates the median, and the whiskers represent the upper and lower adjacent values. Circles indicate points that fall beyond the whiskers. C, Shaded areas indicate 95% CIs. PCIT-AI, artificial intelligence–enhanced parent-child interaction therapy; PCIT-TAU, treatment as usual parent-child interaction therapy.

The differences in ECBI intensity subscale score percentage change (13.86%; 95% CI, −1.41% to 29.14%), ECBI problem subscale score percentage change (38.59%; 95% CI, −1.22% to 78.40%), or absolute change in PSQ scores (−0.16; 95% CI, −1.85 to 1.53) when comparing PCIT-AI with PCIT-TAU did not reach statistical significance (eTable 1 in Supplement 2). Additional sensitivity analyses for ECBI and PSQ changes comparing PCIT-AI to PCIT-TAU when ASD samples were removed from the PCIT-AI arm and intention-to-treat samples were included but did not reach statistical significance (eTables 2 and 3 the Supplement 2). Parents in the PCIT-AI and PCIT-TAU arms recorded a total of 573 and 359 tantrums, respectively. In the PCIT-AI arm, 326 tantrums (57%) were logged after receiving platform-generated alerts for potential impending tantrums. The median (IQR) time from when the alert was generated to when parents responded to it was 3.65 (2.07-7.08) seconds. The remaining 247 of the 573 tantrums (43%) were reported by the parent independent of the tantrum alert. As shown in Figure 2A, the mean (SD) duration of tantrums was significantly lower in the PCIT-AI arm than in the PCIT-TAU arm (10.4 [20.8] minutes vs 22.1 [30.0] minutes; *P* < .001). Between-group differences remained significant after stratification by CDI sessions of PCIT (Figure 2B) and across all PCIT sessions (Figure 2C). PCIT-AI was associated with a significantly lower risk of tantrums lasting 15 minutes or more (OR, 3.66; 95% CI, 2.72-4.95) and 25 minutes or more (OR, 3.30; 95% CI, 2.30-4.90) compared with PCIT-TAU (eTable 4 in Supplement 2).

## Discussion

This study demonstrated the feasibility, adherence, and utilization of a real-time DT augmentation of PCIT for young children with disruptive behaviors. Smartwatch wear times (adherence) exceeded 75% in this naturalistic treatment-seeking sample, thus achieving the feasibility benchmark and primary end point of this trial. Parental responses to alerts of impending tantrums in the PCIT-AI arm were rapid, requiring a median of less than 4 seconds, thus indicating a high level of adherence to cues designed to enable proactive PCIT-driven parental interventions. The current findings suggest that DT augmentations of PCIT are associated with high levels of participant and parental adherence and that these interventions may facilitate successful proactive parenting behaviors using real-time alerts for impending tantrums.

This is the first study, to our knowledge, to demonstrate reduction of tantrum durations reported by parents using digital, ecologic, momentary assessments (as opposed to retrospective recall with traditional rating scales), supported by real-time alerts generated using smartwatches. This technology may address an important clinical need by serving as an early-warning system, enabling the parental application of evidence-based PCIT principles and practices before or at the earliest stages of potentially severe emotional outbursts in children who struggle with disorders characterized by severe episodes of emotional and behavioral dyscontrol. The adherence to digital technology combined with the study completion rate (74%) is promising given that dropout rates in PCIT clinical trials are nearly 50%.^30,31^ With the increasing calls for the use of evidence-based interventions for children with disruptive behaviors,^4,6^ DTs using readily available wearables could serve as a relatively simple, scalable, and low-touch companion to evidence-based psychotherapies targeting episodic behavioral dysregulation, including internet-delivered therapies for patients and families that reside in communities with limited pediatric mental health infrastructure, transportation challenges, and other barriers to establishing needed cares.

From a methodological viewpoint, despite behavior improvements falling short of statistical significance, the effect size of the improvements in ECBI (after adjusting for covariates) with PCIT-AI seems especially promising given that participants in the comparator arm received the same standard of care intervention. The bar for achieving a statistically significant result was likely raised due to a combination of small size, adjustment for covariates, and the administration of an active and effective treatment intervention in both study groups, consistent with our desire to provide a rigorous preliminary test of DT-augmented PCIT. These observations, in addition to high observed child and parental adherence to the intervention and demonstrated success regarding the blinding of study clinicians and participants, provide strong support for a definitive, well-powered comparative-effectiveness randomized clinical trial of DT-augmented PCIT.

### Limitations

There are limitations to this study. The study smartwatch used is not cleared by the US Food and Drug Administration for pediatric populations. Even with the smallest available band, the device remains oversized for a child’s wrist, which affects sensor placement and data validity. The decision to use a readily available smartwatch was based on issues of cost and access for pediatric mental health. Future studies should evaluate adherence and efficacy when using medical-grade wearables for pediatric populations. Although the study strived for equiprobable assignments of PCIT-AI vs PCIT-TAU by using simple randomization, there was a sample imbalance (Table) because the PCIT-AI arm had more patients with ASD. It is difficult to determine whether this imbalance may have selectively affected the odds of positive outcomes for the PCIT-TAU arm. Future studies evaluating the efficacy and efficiency of digital augmentation of PCIT should use balanced trial designs accounting for comorbid diagnosis (eg, ASD) and social determinants of health. Medication use was observed in more than half of study participants, which may have also resulted in residual biases with potential impacts that are difficult to determine. This study was not specifically designed to establish or delineate medication-dependent effects on child behavior. The digital alerts in the PCIT-AI arm were based on a surrogate measure of impending distress (mean heart rate within a window of 10 minutes) and could function only when the parent and child were within the wireless signal range. Therefore, some tantrums may have been missed. In addition, there was not an efficient way to account for unwitnessed tantrums or potential inaccuracies in the tantrum logs. This study, which aimed to evaluate the feasibility and effectiveness of a digital augmentation of PCIT, was conducted at a single site, with all therapy sessions provided by a single blinded clinician who provided interventions. A multicenter randomized clinical trial involving multiple PCIT providers is now warranted to assess the broader effectiveness of and generalization of adherence to digital augmentation of PCIT with wearables. The results presented in this report are from complete case analyses, and the impact of noncompletion on effectiveness of DTs for managing children diagnosed with disruptive behavior is unclear due to limited adherence from early dropout.

## Conclusions

To our knowledge, this study provides the first demonstration of the feasibility of DT augmentation of behavior therapy involving parents and young children with disruptive behaviors. The current findings suggest that digital augmentations of PCIT are associated with high levels of participant and parental adherence and that these interventions may facilitate successful proactive parenting behaviors using real-time alerts for impending tantrums. The results of this study in which both comparator groups received standard-of-care PCIT provide a strong basis for larger, well-powered randomized comparative effectiveness trials of DT augmentation for children with disruptive behavioral disorders.

## Notes

Supplement 1. Trial Protocol

Supplement 2. **eAppendix 1. **Description of the Tantrum Log Programmed in Ilumivu Mobile Application **eAppendix 2. **Technology Support **eAppendix 3. **Adverse Reactions and Technology Reports **eTable 1.** Secondary Outcomes: Behavior and Sleep **eTable 2. **Secondary Outcomes for Intent to Treat Samples **eTable 3. **Secondary Outcomes for Completers, Excluding ASD Samples From the PCIT-AI Arm **eTable 4. **Exploratory Outcomes: Tantrum Durations **eFigure 1. **The Kaplan-Meier Plot of Dropout by Treatment Arm **eFigure 2. **Variation of Mean Adherence (% Time Smartwatch Worn per Day) During Study Period Stratified by Child and Participating Parent

Supplement 3.
Data Sharing Statement
