# Supplementary material for: Feasibility of Digital Augmentation of Parent-Child Interaction Therapy: A Randomized Clinical Trial
Source: JAMA Netw Open. 2025 Dec 15;8(12):e2548869. doi: 10.1001/jamanetworkopen.2025.48869 (PMC12706678; doi:10.1001/jamanetworkopen.2025.48869)
Supplement: Supplement 1. — Trial Protocol [file jamanetwopen-e2548869-s001.pdf]

Supplemental Online Content

Romanowicz M, Saliba MT, Wilton AR, et al. Feasibility of digital augmentation of parent-child interaction therapy: a randomized clinical trial. *JAMA Netw Open*. 2025;8(12):e2548869. doi:10.1001/jamanetworkopen.2025.48869

eAppendix 1. Description of the Tantrum Log Programmed in Ilumivu Mobile Application
eAppendix 2. Technology Support
eAppendix 3. Adverse Reactions and Technology Reports
eTable 1. Secondary Outcomes: Behavior and Sleep
eTable 2. Secondary Outcomes for Intent to Treat Samples
eTable 3. Secondary Outcomes for Completers, Excluding ASD Samples From the PCIT-AI Arm
eTable 4. Exploratory Outcomes: Tantrum Durations
eFigure 1. The Kaplan-Meier Plot of Dropout by Treatment Arm
eFigure 2. Variation of Mean Adherence (% Time Smartwatch Worn per Day) During Study Period Stratified by Child and Participating Parent

This supplemental material has been provided by the authors to give readers additional information about their work.

© 2025 Romanowicz M et al. *JAMA Network Open.*

eAppendix 1. Description of the Tantrum Log Programmed in Ilumivu Mobile Application

**Survey Name**	**Tantrum Log**
**Survey Description**	This ecological momentary assessment logs the time of start and end of a tantrum, provided by parent on demand.
**Survey Frequency**	On demand by parent
**Recipients**	All participating parents in the study.
**Survey**	1. Time of Onset of Tantrum<br>2. End Time of Tantrum<br><br>Time reported as DD/MM/YYYY HH:MM

eAppendix 2. Technology Support

The study team provided support to 1) setup the smartwatches for the dyad at enrollment, 2) prepare the Ilumivu application on the respective mobile devices, and 3) manage an 18-hour (6am to midnight) study-dedicated email that participants were asked to email should they encounter any technical difficulties. Solutions to any technical issues were resolved via email, a phone call with a study team member, or in-person before or after a PCIT session. Participants were asked to not wear the watch if there was any skin irritation (an anticipated adverse reaction) in the wrist area and encouraged to resume wearing the watch when deemed comfortable.

eAppendix 3. Adverse Reactions and Technology Reports

Three children (PCIT-AI: n = 1; PCIT-TAU: n = 2) reported a minor skin rash in the wrist area which resolved within a week by parents' care. No adverse reactions were reported by any of the participating parents. A total of 26 smartwatches were replaced (children = 19; parents = 7) due to Bluetooth connectivity issues (n = 2), battery not holding charge (n = 7), hardware glitches (n = 1), lost (n=5), and damaged watch bands (n=11). Upon completion, parents reported the need for frequent charging of the study-issued iPods (all 36 issuances of iPods) installed with only the Ilumivu mobile application. However, the same application installed in the study-issued (n=31) iPhone 11 did not result in any report of reduced battery charge retention.

© 2025 Romanowicz M et al. *JAMA Network Open.*

eTable 1. Secondary Outcomes: Behavior and Sleep

Measure	Estimated difference*,#	95% CI	p-value	Cohen's d Effect size
Percent change in ECBI: intensity	13.86	(-1.41, 29.14)	0.07	0.37
Percent change in ECBI: problem	38.59	(-1.22, 78.40)	0.06	0.20
Absolute Change in PSQ	-0.16	(-1.85, 1.53)	0.85	0.11

*Difference in secondary outcomes between PCIT-AI and PCIT-TAU (a positive estimated difference indicates a numerically higher improvement than PCIT-TAU). #Adjusted for baseline score, child age, child sex, and indicator of child with ADHD diagnosis taking either stimulants or non-stimulants.

eTable 2. Secondary Outcomes for Intent to Treat Samples

Measure	Estimated difference*,#	95% CI	p-value	Cohen's d Effect size
Percent change in ECBI: intensity	5.99	(-9.30, 21.28)	0.43	0.22
Percent change in ECBI: problem	15.47	(-20.05, 51.00)	0.38	0.15
Absolute Change in PSQ	-0.08	(-1.60, 1.44)	0.91	0.02

*Difference in secondary outcomes between PCIT-AI and PCIT-TAU (a positive estimated difference indicates a numerically higher improvement than PCIT-TAU). #Adjusted for baseline score, child age, child sex, and indicator of child with ADHD diagnosis taking either stimulants or non-stimulants.

eTable 3. Secondary Outcomes for Completers, Excluding ASD Samples From the PCIT-AI Arm

Measure	Estimated difference*,#	95% CI	p-value	Cohen's d Effect size
Percent change in ECBI: intensity	15.36	(-2.34, 3.07)	0.09	0.44

© 2025 Romanowicz M et al. *JAMA Network Open.*

Percent change in ECBI: problem	32.03	(-11.09, 75.15)	0.14	0.12
Absolute Change in PSQ	1.00	(-1.38, 3.38)	0.40	0.06

*Difference in secondary outcomes between PCIT-AI and PCIT-TAU (a positive estimated difference indicates a numerically higher improvement than PCIT-TAU). #Adjusted for baseline score, child age, child sex, and indicator of child with ADHD diagnosis taking either stimulants or non-stimulants.

eTable 4. Exploratory Outcomes: Tantrum Durations

	PCIT-AI	PCIT-TAU	p-value
**Total logged tantrums, n (%)**	573	359	
Duration of tantrums (mins), mean (SD)	10.4 (20.8)	22.1 (30.0)	<0.001
Duration of tantrums during CDI (mins), mean (SD)	10.7 (23.9)	22.3 (32.4)	<0.001
Duration of tantrums during PDI (mins), mean (SD)	9.8 (9.8)	21.5 (23.8)	0.12
**Total tantrums lasting ≥15 mins**	120	177	
Duration of tantrums during CDI (mins), mean (SD)	31.4 (45.5)	40.3 (41.2)	0.29
Duration of tantrums during PDI (mins), mean (SD)	29.1 (13.0)	31.8 (26.8)	0.89
**Total tantrums lasting ≥25 mins**	55	94	
Duration of tantrums during CDI (mins), mean (SD)	49.6 (64.9)	55. (47.3)	0.55
Duration of tantrums during PDI (mins), mean (SD)	39.3 (10.2)	53.3 (33.1)	0.29

© 2025 Romanowicz M et al. *JAMA Network Open.*

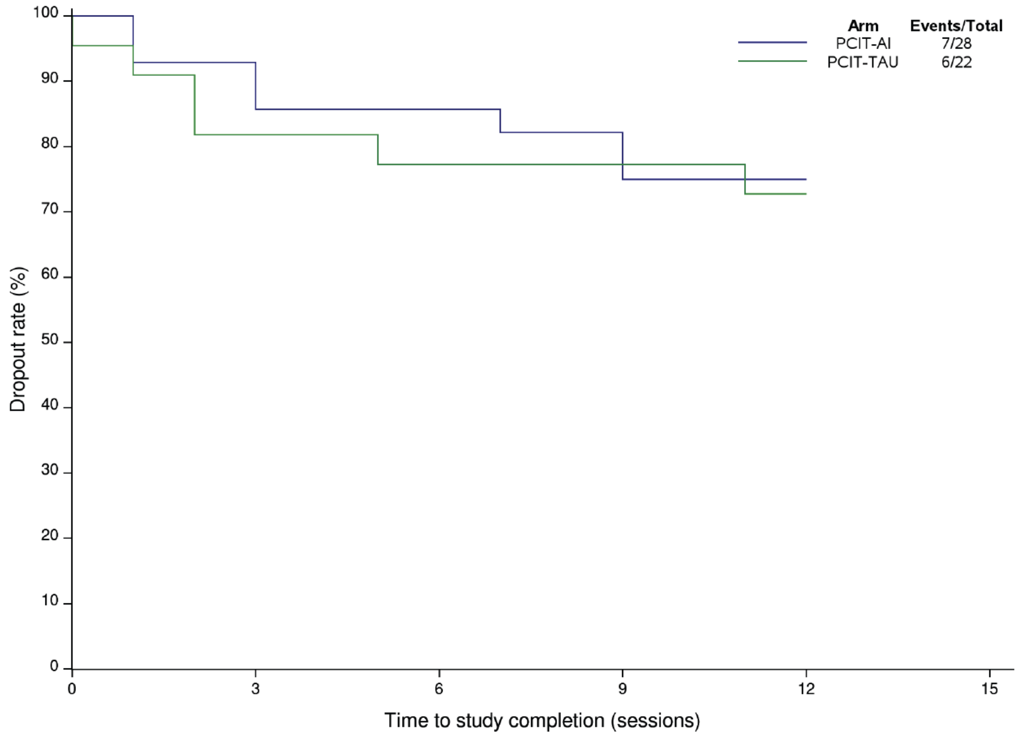

Patients-at-Risk

PCIT-AI	28	26	24	24	23	21	21
PCIT-TAU	22	20	18	17	17	17	16

eFigure 1. The Kaplan-Meier Plot of Dropout by Treatment Arm

© 2025 Romanowicz M et al. *JAMA Network Open.*

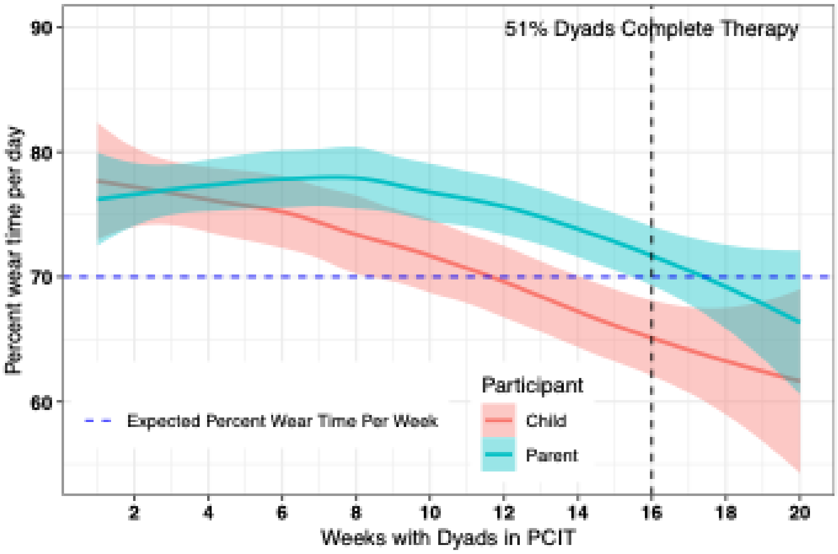

eFigure 2. Variation of Mean Adherence (% Time Smartwatch Worn per Day) During Study Period Stratified by Child and Participating Parent

The shaded region is the 95% confidence interval of adherence.

© 2025 Romanowicz M et al. *JAMA Network Open.*
