# Supplementary material for: Feasibility of Digital Augmentation of Parent-Child Interaction Therapy: A Randomized Clinical Trial
Source: JAMA Netw Open. 2025 Dec 15;8(12):e2548869. doi: 10.1001/jamanetworkopen.2025.48869 (PMC12706678; doi:10.1001/jamanetworkopen.2025.48869)
Supplement: Supplement 2. — eAppendix 1. Description of the Tantrum Log Programmed in Ilumivu Mobile Application eAppendix 2. Technology Support eAppendix 3. Adverse Reactions and Technology Reports eTable 1. Secondary Outcomes: Behavior and Sleep eTable 2. Secondary Outcomes for Intent to Treat Samples eTable 3. Secondary Outcomes for Completers, Excluding ASD Samples From the PCIT-AI Arm eTable 4. Exploratory Outcomes: Tantrum Durations eFigure 1. The Kaplan-Meier Plot of Dropout by Treatment Arm eFigure 2. Variation of Mean Adherence (% Time Smartwatch Worn per Day) During Study Period Stratified by Child and Participating Parent [file jamanetwopen-e2548869-s002.pdf]

PISTACHIO (PREEMPTIION OF DISRUPTIVE BEHAVIOR IN CHILDREN) REAL-TIME MONITORING OF SLEEP AND BEHAVIOR OF CHILDREN 3-7-YEAR-OLD RECEIVING PARENT CHILD INTERACTION THERAPY AUGMENTED WITH ARTIFICIAL INTELLIGENCE RANDOMIZED CONTROLLED TRIAL.

Principal Investigators:

Magdalena Romanowicz, MD
Department of Psychiatry and Psychology
Mayo Clinic
200 First Street SW
Rochester, MN 55905
(507) 255-6782 (office)
(507) 293-6089 (pager)
(507) 269-3375 (cell phone)
romanowicz.magdalena@mayo.edu

Arjun Athreya, PhD, MS
Department of Pharmacology
Mayo Clinic
200 First Street SW
Rochester, MN 55905
(507) 422-6073
athreya.arjun@mayo.edu

Paul Croarkin, DO
Department of Psychiatry and Psychology
Mayo Clinic
200 First Street SW
Rochester, MN 55905
(507) 293-5413
croarkin.paul@mayo.edu

Co-Investigators:

Julia Shekunov, MD
Department of Psychiatry and Psychology
Mayo Clinic
200 First Street SW
Rochester, MN 55905
(507) 292-4052
shekunov.Julia@mayo.edu

Funding Sponsor: Mayo Clinic

Protocol Number: Mayo Clinic IRB# 21-007403

Version Protocol Date: 2/17/2023

Version No.	Date	Major Changes
1	6/15/2021	Initial
2		IRB Specialist Changes
3		IRB deferral changes
4	2/9/2022	Protocol updates
5	4/5/2022	Changing the frequency of surveys and timeframe of blood draw
6	8/2/2022	Adding Parental Exit Interview
7	3/10/2023	Adding Bluetooth requirements
8	8/8/2023	Return of CRP blood results to patients

Table of Contents

STUDY SUMMARY **6**

1. INTRODUCTION **9**

1.1 BACKGROUND 9
1.2 STUDY RATIONALE AND RISK ANALYSIS 10
1.2.1 Study Rationale *10*
1.3 CLINICAL DATA TO DATE 11
1.4 RISKS AND BENEFITS 13
1.4.1 Clinical Assessments and Testing *13*
1.4.2 Potential Benefits *14*
1.5 ANTICIPATED DURATION OF THE CLINICAL INVESTIGATION 14

2. STUDY OBJECTIVES **14**

3. STUDY DESIGN **16**

4. PARTICIPANT SELECTION, ENROLLMENT AND WITHDRAWAL **18**

4.1 INCLUSION CRITERIA 18
4.2 NUMBER OF SUBJECTS 19
4.3 DURATION OF PARTICIPATION 19
4.4 PARTICIPANT RECRUITMENT, ENROLLMENT, SCREENING, STUDY PROCEDURES AND FOLLOW-UP 19
4.4.1 Recruitment *19*
4.4.2 Pre-screening *22*
4.4.3 Consent/Assent *22*
4.4.4 Screening *22*
4.4.5 Study Procedures *23*
4.5 EARLY WITHDRAWAL OF PARTICIPANTS 23
4.5.1 Participant Withdrawal, Data Collection, and Follow-Up *23*

5 STUDY PROCEDURES, DEVICES, AND INSTRUMENTS. **24**

5.1 PRE-SCREENING 24
5.2 STUDY VISIT 1 24
5.2.1 Part 1- Screening *24*
5.2.2 C - reactive protein (CRP) *25*
5.2.3 Plasma Multi-Omics *25*
5.2.4 Part 2- Smartwatch measures *25*
5.2.5 Part 3- Psychiatric Interview and Questionnaires *26*
5.3 POST CDI INTERVAL ASSESSMENT VISIT 27
5.4 OUTCOME ASSESSMENT VISIT 28
5.5 PCIT TREATMENT 28
5.6 BIOMARKER SAMPLE COLLECTIONS 29

COLLECTIONS **29**

BLOOD COLLECTION **29**

6. STATISTICAL PLAN **33**

6.1 STUDY PURPOSE 33
6.2 STATISTICAL METHODS 33
6.3 SAMPLE SIZE DETERMINATION 33
6.4 DATA ANALYSIS PLAN FOR AIM 1 33
6.5 DATA ANALYSIS PLAN FOR AIM 2 33

7. TREATMENT AND SAFETY PLAN 34

7.1 TREATMENT 34
7.2 SAFETY PLAN 35
7.3 DATA AND SAFETY MONITORING 35
7.3.1 Subject Safety 35
7.3.2 Subject Removal from Study 36
7.3.3 Reporting Mechanisms 36
7.3.4 Data Integrity 36
7.3.5 Subject Privacy 36
7.3.6 Data Confidentiality 36
7.3.7 Product Accountability 36
7.3.8 Study Documentation 37
7.3.9 Study Coordination 37

8. DATA HANDLING AND RECORD KEEPING 37

8.1 CONFIDENTIALITY 37
8.2 SOURCE DOCUMENTS 37

9. STUDY FINANCES 38

9.1 FUNDING SOURCE 38
9.2 PARTICIPANT STIPENDS OR PAYMENTS 38

PUBLICATION PLAN 38

10. REFERENCES 38

LIST OF ABBREVIATIONS

AE	Adverse Event/Adverse Experience
AI-PCIT	Artificial Intelligence Augmented Parent Child Interaction Therapy
CBCL	The Child Behavior Checklist
CCNES	Coping with Children's Negative Emotions
CDI	Child Directed Interaction
CFR	Code of Federal Regulations
CGAS	Children's Global Assessment Scale
CGI-I	Clinical Global Impression – Global Improvement
CRF	Case Report Form
CRP	C-reactive P
Dem	Demographics
EBP	Externalizing Behavioral Problems
ECBI	Eyberg Child Behavior Inventory
FDA	Food and Drug Administration
FH	Family History
HR	Heart Rate
GCP	Good Clinical Practice
HIPAA	Health Insurance Portability and Accountability Act
IB	Investigator's Brochure
IRB	Institutional Review Board
K-SADS-EC	Kiddie-Schedule for Affective Disorders and Schizophrenia-Early Childhood
Med	Medications
PAPA	Preschool Age Psychiatric Assessment
PCIT	Parent Child Interaction Therapy
PCIT-sham	Parent Child Interaction Therapy-sham
PDI	Parent Directed Interaction
PECFAS/CAFAS	Preschool and Early Childhood Functional Assessment Scale/Child and Adolescent Functional Assessment Scale
PFC	The Preschool Feelings Checklist
PHI	Protected Health Information
PI	Principal Investigator
PPH	Past Psychiatric History
PSI-SF	Parenting Stress Index-Short Form
PSQ	Pediatric Sleep Questionnaire
SAE	Serious Adverse Event/Serious Adverse Experience
SH	Social History
TAI	The Therapy Attitude Inventory

Study Summary

Title	PISTACHIO (PREEMPTIION OF DISRUPTIVE BEHAVIOR IN CHILDREN) REAL-TIME MONITORING OF SLEEP AND BEHAVIOR OF CHILDREN 3-7-YEAR-OLD RECEIVING PARENT CHILD INTERACTION THERAPY AUGMENTED WITH ARTIFICIAL INTELLIGENCE RANDOMIZED CONTROLLED TRIAL.
Running Title	PISTACHIO (PREEMPTIION OF DISRUPTIVE BEHAVIOR IN CHILDREN)
Protocol Number	Mayo Clinic IRB# 21-007403
Phase	Phase I, exploratory and feasibility study
Methodology	Double blind, Randomized controlled trial
Overall Study Duration	2 years
Subject Participation Duration	The estimated length of the PCIT treatment from start to finish is about 12 weekly 50 min long sessions (6CDI sessions and 6PDI sessions). Participants will be asked to participate in 2 hrs long assessment visit pre and post treatment.
Single or Multi-Site	Single center Rochester MN

Objectives	**Aim 1:** To evaluate feasibility of the use of Garmin wearables in children aged 3-7 years with emotional behavioral problems (EBP).<br><br>**Hypothesis 1:** Children with EBP undergoing PCIT will adhere to and tolerate wearables (e.g., Garmin vivosmart4 smartwatch) as evidenced by at least 70% of enrolled patients being able to wear it for 70% of their treatment period as evidenced by sleep, heart rate and intensity of activity data obtained from Garmin watches.<br><br>**Aim 2a:** To evaluate effectiveness of AI-PCIT by comparing weekly behavioral treatment outcomes as measured by ECBI in AI-PCIT group as compared with PCIT- sham biometric group over a 12-week period.<br><br>**Hypothesis 2a:** Children in AI-PCIT group will have a greater mean reduction of ECBI scores than children in PCIT- sham biometric in comparison to PCIT sham biometric group over a 12-week period.<br><br>**Aim 2b:** To evaluate effectiveness of AI-PCIT by comparing weekly sleep treatment outcomes as measured by PSQ in AI-PCIT group as compared with the PCIT- sham biometric group over of 12-week period.<br><br>**Hypothesis 2b:** Children in AI-PCIT group will demonstrate greater improvements in sleep as assessed by mean PSQ scores in comparison to the PCIT sham biometric group over a 12-week period.<br><br>**Aim 3a:** To evaluate concordance of smartwatch measurements of sleep data from young patients as compared to parental rating scales of PSQ (obtained weekly).<br><br>**Hypothesis 3a:** Sleep cycle data (deep sleep, light sleep, rem and awake) from smartwatches indicative of sleep quality will correlate with parental reports on sleep (PSQ) obtained weekly.<br><br>**Aim 3b:** To evaluate accuracy of Indirect Behavioral Measures from smartwatches (i.e., measurements of heart rate, intensity of activity) from young patients as compared to parental rating scales of ECBI (obtained weekly).<br><br>**Hypothesis 3b:** Indirect Behavioral Measures from smartwatches that monitor the heartrate variability and intensity of activity (i.e., is the child sedentary or active or highly active) will identify episodes of disruptive behavior that will be reflective of parental reports on behavior (ECBI) obtained weekly.

	**Exploratory aim:** To examine a link between Garmin biometric measurements of HR, levels of activity sleep, and outcome of PCIT as assessed with CRP values (blood) obtained before and after treatment).<br><br>**Exploratory Hypotheses:** Baseline CRP levels in child participants will demonstrate direct correlations with ECBI scores, average heart rate and activity levels. Baseline CRP levels with have an indirect correlation with sleep quality assess with the Garmin wearable throughout the trial. Mean baseline CRP levels will have an indirect correlation with mean change in ECBI over 12 weeks of treatment. An exploratory aim will collect plasma from both the child and the participating parent to characterize multi-omic associations of disruptive behaviors in a parent-child dyad study.
Number of Subjects	25 patients and 25 parents enrolled to the active arm of the study (AI-PCIT). 25 patients and 25 parents enrolled to the control group (PCIT-sham biometric). N = 100.
Diagnosis and Main Inclusion Criteria	Main diagnoses: Problem behavioral child, emotional behavioral dyscontrol, Attention deficit hyperactivity disorder, Oppositional defiant disorder. Inclusion criteria include children ages 3-7 years old whose measure of EBP rated above the clinically significant range (≥120; T-score ≥ 60) (Eyberg Child Behavior Inventory- ECBI; Eyberg & Pincus, 1999). Families approached for participation will be asked to commit to entire 12-week, outpatient treatment course of PCIT, and at least one primary caregiver and the identified child will have to be able to speak and understand English.
Reference therapy	Parent Child Interaction Therapy (PCIT) is evidence based behavioral parent training therapy that helps parents learn specialized skills to increase their child's prosocial behavior and decrease aggressive and noncompliant behavior. This treatment typically takes 12 weekly sessions, if attended consistently.
Statistical Methodology	The primary goal of the study is to establish feasibility of the proposed augmentations to PCIT. This study is an exploratory clinical trial with the broad goals of demonstrating feasibility, generating pilot data, and to generate validate effect sizes, to inform future larger, multicenter trials. The 3 primary hypotheses will be examined with descriptive statistics, mixed linear regression models, and Spearman correlation coefficients. The multi-omic measures are for a descriptive, hypothesis generating aim with no formal hypothesis.

1. Introduction

This document is a protocol for a human research study. This study will be carried out in accordance with the applicable United States government regulations and Mayo Clinic research policies and procedures.

1.1 Background

It is estimated that nearly 25% of preschool-aged children struggle with psychosocial stress and social-emotional issues.(1) Between 15 and 34% of same aged children are reported to have significant early externalizing behavior problems (EBP) such as aggression, oppositional behaviors, problems with concentration and impulsivity,(2) as measured by ECBI (Eyberg Child Behavior Inventory).(3) It is particularly concerning as if these symptoms that emerge at a very young age are untreated, ongoing impairments persist throughout development and may lead to negative outcomes such as academic and legal difficulties, violent behaviors and significant mental health issues and substance abuse.(4-6) Early intervention programs that are evidence-based and easy to implement are crucial in helping young children with EBP. (7)

The challenge: As of today, parents often have difficulty recollecting the events (number, timing of tantrums, precipitating events) even from the week before in their young children. They also are frequently unsure how their child slept. Rating scales assist parents with the recall, but they are not always reliable. Providers rely on a brief period of observation of the child in an artificial environment of their office that is not always reflective of the child's behavior at home and/or school. These factors contribute to suboptimal outcomes. It is unfortunate as EBP is common, starts early in life and often may lead to significant disabilities. (8) Our ongoing program of research and the present study proposes development of wearable technology, such as Garmin coupled with artificial intelligence platforms for reliable and valid data collection to advance treatment protocols and outcomes. This in turn would enable the clinician to design an individualized treatment plan based on real-time information about the child's behavior. Recently, we completed pilot work with Garmin wearable for comparisons with standard of care practices on our inpatient psychiatric unit that allowed for 24 hours observation of the child's behavior. The next incremental step is to integrate and study the use of these tools in outpatient practice. This study focuses on the Young Child Behavioral Clinic were patients aged 3-7 years receive Parent Child Interaction Therapy (PCIT).

PCIT is one of the four Behavior Parent Training (BPT) programs that are considered "well supported by research evidence" by the California Evidence-Based Clearinghouse for Child Welfare (http://www.cebc4cw.org).(8) It is a dyadic parent-child therapy with unique elements of in-vivo parent coaching via bug in the ear by the therapist who works from behind a one-way mirror. Most important PCIT allows for individually tailored coaching that can address unique difficulties of particular parent-child dyads. A recent meta-analysis that studied effectiveness of PCIT with the main focus on modifications, study design, and bias showed that PCIT was not only superior in comparison with control groups for reducing externalizing behaviors in children but also helped with parent and child-related stress. (9) The review also showed that PCIT

effectiveness was not affected by session length, various settings (academic versus community), or presenting problems (disruptive behaviors alone as compared to other problems complicated by disruptive behaviors). Additionally, PCIT's unique focus on mastery-based therapy progression makes the components of PCIT ideal for modifications. (9)

Despite PCIT effectiveness, high dropout rate (in some studies as high as 50%) prior to completion of the treatment remains a significant problem.(10) Currently there are some efforts to reduce high attrition by the use of new technologies such as tele-health to remotely deliver real-time therapy to the patient's home. (11)The current study aims to examine and facilitate further development of an innovative Artificial Intelligence PCIT (AI-PCIT) protocol augmented with wearable technologies such as Garmin.

Wearable technologies such as Garmin smartwatches allow for objectively measuring activities that extend beyond observations reported in the clinical settings. Garmin smartwatches have been tested for continuous monitoring during pregnancy(12) and promoting physical activity in adolescents with juvenile idiopathic arthritis. (13) Despite the clinical need, wearable technologies have not been widely studied nor implemented in for the treatment of young children with EBP.

In our study we will also measure C-reactive protein (CRP) as main biomarker in children and parents. CRP is a known marker of chronic inflammation, at high levels it alerts to acute infection, however at minor elevations to points to stress-related immune dysregulation and has been associated with psychosocial stress.(14) Much is known about effects of elevated CRP levels in adulthood on long term medical and psychiatric issues (chronic stress) however little is known whether CRP elevations start in early life.(15) To date there has been only one study that explored CRP in preschoolers that showed no significant correlations between mother's chronic inflammation and child inflammation.(16) They hypothesized that it might be difficult to detect chronic inflammation in child as young as 3 y.o. however this was only a pilot study. Other studies were able to show strong associations between CRP levels and early childhood stress in children as young as 10 and persist into adulthood.(17)

At the biological level, complex psychiatry behaviors are better characterized by studying the interaction of environmental factors (i.e., exposome) and body's internal factors (e.g., genome, metabolome and proteome).(19) Hence, this study will collect plasma from both the child and the participating parent to characterize multi-omic associations of disruptive behaviors in a parent-child dyad study.

1.2 Study rationale and Risk Analysis

1.2.1 Study Rationale

The project's intellectual merit is timely and significant. PISTACHIo will be the first individualized digital biomarker-based technology to remotely reinforce positive engagement

with children diagnosed with emotional behavioral disorders. By virtue of preempting behavior outbursts and monitoring parental response via a mobile-based ecological momentary assessment, PISTACHIo will generate new knowledge on parental behavior and response to effectively managing their child's behavior. Through collaborations with social workers, psychologists, psychiatrists at Mayo Clinic the project will generate a broad three shields development plan that also enumerates guidelines for international knowledge translation, education, action, adoption, and implementation of PISTACHIo at broader community levels.

We will recruit 50 participants between age 3-7 years old whose measure of EBP rated above the clinically significant range (T-score ≥ 60) (Eyberg Child Behavior Inventory- ECBI; Eyberg & Pincus, 1999)(3) who will be randomly assigned to AI-PCIT group or PCIT- sham biometric group. Each child will be recruited with one of their parents ages 18-99 for a total recruitment of 100 participants. Patients eligible for PCIT treatment will be approached by the study team for pre-screening. A pre-screening interview will be carried out to determine initial eligibility. Each participant will undergo a review of inclusion and exclusion criteria.

Children and parents who qualify and agree to participate in the study will be given Garmin devices during their CDI-teach session. Data will be stored and used at the end of the study. Data from Garmin smartwatches are routinely synced to Garmin Connect via Ilumivu or Fitabase, which can then be updated for the research study during the patient's weekly visits. Each child's mother or primary caregiver will also be given a Garmin to collect biometric data for exploratory, hypothesis generating data collection.

The current study aims to develop an innovative wearable tracking protocol that will use AI technology. This would be first time such treatment model is used in young children below 8 years old and as such our proposal is innovative in number of ways; it has the potential to begin critical work on innovative assessment strategies for the youngest patients struggling with significant emotional- behavioral issues. Garmin data and artificial intelligence approaches will have enhanced temporal resolution compared to standard assessments. Specifically, these innovations will facilitate: 1) collection of more objective and longitudinal behavioral measures, 2) assessments of standard clinical outcome measures such as the ECBI and PSQ, the rate of tantrum events/duration everyday activities, 3) identify early opportunities for reinforcing positive behavior that are child-specific and 4) understand early signatures of child's behavioral difficulties. Artificial intelligence methods such as reinforcement learning and probabilistic graphical models will be used to develop methods to analyze data in this project as they are suitable for longitudinal data that can incorporate time-sensitive changes and physician's domain knowledge to make prognoses sensitive and interpretable.

1.3 Clinical Data to Date

The research team recently completed a feasibility study: IRB 20-002133 "Real-Time Monitoring of Sleep and Behavior of Latency Children on Inpatient Child Psychiatric Unit Augmented with Artificial Intelligence." The study enrolled 11 subjects on our Acute Inpatient Child Psychiatric Unit. All subjects were asked to wear Garmin Vivosmart4 smartwatches and nurses monitored children's behaviors 24hrs during their inpatient stay and logged the child's behavior using a chart. Our main goal was to compare the readings from the Garmin watch against the current standard of care which is direct observation.

The study demonstrated (See Fig. 1), the feasibility of physiology data collection derived from a Garmin smartwatch and potential utility in predicting impending behavioral outburst (e.g., a tantrum). The subject was playful until 6:10pm after dinner and was tasked with completing homework. Although the child was sedentary (S in the text block of figure) for most part of the times, child was likely frustrated with the task (indicated by increased heartrate) and when asked "how are you doing?", the child erupts at 6:48 pm and needed intensive behavioral interventions (seclusion) for their safety and that of others (duration marked by red vertical lines). The trend of the increased heartrate while sedentary is evident nearly 30 minutes prior to becoming disruptive (see Fig. 1B) which summarizes heartrate variations as z-scores across all patients with and without tantrums. Parents and treatment team members warned of this high-chance of disruption could positively engage with the child and comfort them – thereby providing a positive behavioral intervention.

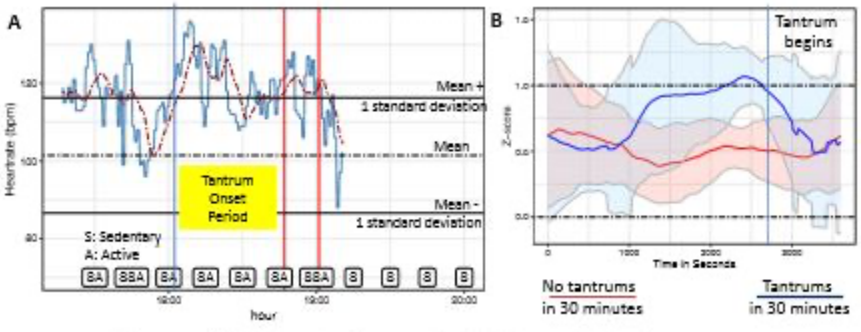

Figure 1: Heart variation and tantrums.

This pilot study also suggested that sleep patterns may predict behavior for the next day (see Fig. 2): Parents often ask if child's sleep affects behavior the following day. The research team observed that duration of light sleep was higher ($p < 0.006$) in children who had behavioral outbursts the following day suggesting the potential for a "weather-app" equivalent prediction of "what is my child's likelihood of behavior disruption today?".

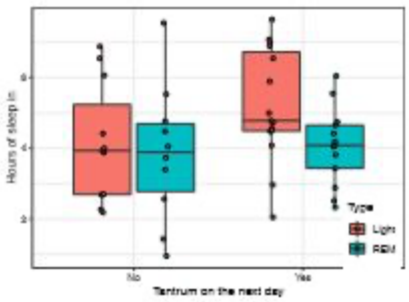

Figure 2: Sleep and tantrums.

1.4 Risks and Benefits

This study is considered minimal risk with the potential of therapeutic benefits to individuals participating in this study. The study patients and parents will all receive clinical standard of care PCIT. All child participant and the primary caretakers will wear a Garmin wearable and undergo clinical research assessments. There is a reasonable expectation, that these augmentations related to study participation will improve the outcomes for participating children and parents in both study arms.

The risks of having blood drawn include pain, bruising, or, rarely, infections at the site of needle stick. These risks are no different than with any routine clinical blood draw. The blood samples provided during this study will be de-identified and used for exploratory biomarker. They will not be stored with any name, address, Mayo Clinic number, birth date, or social security number on them. Instead, samples will be assigned a unique code. Only certain members of the study staff will know what name goes with what code. Although extremely unlikely, there is a risk of a breach in confidentiality.

1.4.1 Clinical Assessments and Testing

The psychiatric assessment, interview, and rating scales in this study are time consuming and may bring up upsetting issues. If suspected child abuse or neglect is detected, the research team is obligated to follow mandatory, federal, state, and institutional reporting laws.

1.4.1.1 Confidentiality

The potential for a breach in confidentiality always exists, specifically with the written research data and study databases. Deidentified data will be entered into a RedCap database with double password protection. However, information that is obtained will be stored in locked file drawers in locked offices; data will have identifying information sheered from it to prevent loss of confidentiality, all computers and databases will be password protected with passwords available to limited study personnel, and all staff must sign confidentiality certificates.

Loss of confidentiality is a potential risk because participants will be asked to disclose their recent medications history, information pertaining to demographics, family history, and psychiatric history. Since code numbers will be given to each participant and data will be stored by code number, the risk of loss of confidentiality will be minimal. Only personnel working on this project will have access to the data. Only data that are pertinent to the study will be collected so that seriousness of loss of confidentiality will be minimized. The alternative to participating in this project is to decline. Participants will be at no risk if they decline to participate.

1.4.2 Potential Benefits

PISTACHIo will remotely gather real-time data from multiple sensors (sleep, activity, intensity, and heartrate) in young children with emotional-behavioral disorders to predict impending tantrums as early as 30 minutes. The project will result in an "action plan" of how to respond to behavior alerts.

To our knowledge this will be the first study investigating the use of wearables in young children with emotional-behavioral issues. Findings will have important future implications in enhancing our understanding of disruptive behaviors and leading to development of novel diagnostic and therapeutic interventions. This pilot project will provide critical pilot data and experience for future federal grant submissions (NIMH R01 and NSF). This study is considered minimal risk without any direct or therapeutic benefits to individuals participating in this study. Participants will receive Parent Child Interaction Therapy Standard Protocol which is an evidence-based treatment for emotional behavioral issues in children 3-7 yo. The overall goal of this study and research program is to improve behavioral and psychiatric treatments for young children. Participants whose clinical conditions necessitate immediate treatment will be referred to the appropriate level of care, and participants will be offered referral to ongoing outpatient mental health care at the conclusion of study participation.

1.5 Anticipated Duration of the Clinical Investigation

It is anticipated that the proposed study will be two years in duration. All participants will be expected to participate in 12 weeks of PCIT therapy. We will also ask them to participate in pre and post-evaluation single study visits. We estimate that each visit is expected to last a total of 2 hrs.

All subjects will be asked to elect whether they want to participate in a blood draw (optional). They will be informed that all the sessions are going to be recorded (audio and video) and that specifically voice of their child is going to be analyzed.

2. Study Objectives

Aim 1: To evaluate feasibility of the use of Garmin wearables in children aged 3-7 years with emotional behavioral problems (EBP).

Hypothesis 1: Children with EBP undergoing PCIT will adhere to and tolerate wearables (e.g., Garmin vivosmart4 smartwatch) as evidenced by at least 70% of enrolled patients being able to wear it for 70% of their treatment period as evidenced by sleep, heart-rate and intensity of activity data obtained from Garmin watches.

Aim 2a: To evaluate effectiveness of AI-PCIT by comparing weekly behavioral treatment outcomes as measured by ECBI in AI-PCIT group as compared with PCIT- sham biometric group over a 12-week period.

Hypothesis 2a: Children in AI-PCIT group will have a greater mean reduction of ECBI scores than children in PCIT- sham biometric in comparison to PCIT sham biometric group over a 12-week period.

Aim 2b: To evaluate effectiveness of AI-PCIT by comparing weekly sleep treatment outcomes as measured by PSQ in AI-PCIT group as compared with the PCIT- sham biometric group over of 12-week period.

Hypothesis 2b: Children in AI-PCIT group will demonstrate greater improvements in sleep as assessed by mean PSQ scores in comparison to the PCIT sham biometric group over a 12-week period.

Aim 3a: To evaluate concordance of smartwatch measurements of sleep data from young patients as compared to parental rating scales of PSQ (obtained weekly).

Hypothesis 3a: Sleep cycle data (deep sleep, light sleep, rem and awake) from smartwatches indicative of sleep quality will correlate with parental reports on sleep (PSQ) obtained weekly.

Aim 3b: To evaluate accuracy of Indirect Behavioral Measures from smartwatches (i.e., measurements of heart rate, intensity of activity) from young patients as compared to parental rating scales of ECBI (obtained weekly).

Hypothesis 3b: Indirect Behavioral Measures from smartwatches that monitor the heartrate variability and intensity of activity (i.e., is the child sedentary or active or highly-active) will identify episodes of disruptive behavior that will be reflective of parental reports on behavior (ECBI) obtained weekly.

Exploratory aim: To examine a link between Garmin biometric measurements of HR, levels of activity sleep, and outcome of PCIT as assessed with CRP values (blood) obtained before and after treatment).

Exploratory Hypotheses: Baseline CRP levels in child participants will demonstrate direct correlations with ECBI scores, average heart rate and activity levels. Baseline CRP levels with have an indirect correlation with sleep quality assess with the Garmin wearable throughout the

trial. Mean baseline CRP levels will have an indirect correlation with mean change in ECBI over 12 weeks of treatment. An exploratory aim will collect plasma from both the child and the participating parent to characterize multi-omic associations of disruptive behaviors in a parent-child dyad study.

3. Study Design

We will recruit 50 participants between age 3-7 years old whose measure of EBP rated above the clinically significant range (T-score ≥ 60) (Eyberg Child Behavior Inventory- ECBI; Eyberg & Pincus, 1999)(3) who will be randomly assigned to AI-PCIT group or PCIT- sham biometric group. Each child will be recruited with their parents age 18-99 for a total recruitment of 100 participants. Patients eligible for PCIT treatment will be approached by the study team for pre-screening. A pre-screening interview will be carried out to determine initial eligibility. Each participant will undergo a review of inclusion and exclusion criteria. Protected health information collected during screening of prospective subjects who do not subsequently sign the consent form and/or the HIPAA authorization form will be discarded. Caregivers will be interviewed using the Kiddie-Schedule for Affective Disorders and Schizophrenia-Early Childhood (K-SADS-EC) to assess the child's psychiatric symptoms and assign DSM-5 diagnoses. We will also use Preschool Age Psychiatric Assessment (PAPA), a reliable measure of Axis I disorders (and severity) in preschool children, (Egger, et al., 1999; Egger et al.,2006). Children's Global Assessment Scale (CGAS) will be completed by the clinician-rater to measures children's global level of impairment. Parenting Stress Index (PSI) will be used to measure the magnitude of stress within the parent-child dyad via caregiver report. Coping with Children's Negative Emotions (CCNES) will be used to assess parental coping styles and strategies in response to children's expression of negative emotions via caregiver report. CBCL will be obtained to capture a wider range of problematic behaviors. We will also obtain Preschool Feelings Checklist (PFC) to quickly screen for presence of any symptoms of depression in our population. The Pediatric Sleep Questionnaire (PSQ) will be used to examine sleep behavior in young children. The Therapy Attitude Inventory (TAI) will be used to measure treatment satisfaction. Trauma Symptom Checklist for Young Children (TSCYC) will be used for the assessment of trauma-related symptoms in children ages 3-12. Early Childhood Screening Assessment (ECSA) will be used to identify very young children (1½-5 years old) who need further emotional or behavioral assessment. Clinical Global Impression – Global Improvement (CGI-I) will be used to measure the blind clinician-rater's impression of improvement at post assessment. Dyadic Parent-Child Interaction Coding System II (DPICS-II) is a coding system for specific structured parent-child interactions targeted by PCIT.

Children and parents who qualify and agree to participate in the study will be given Garmin devices during their screening/baseline assessment visit. Data will be stored and used at the end of the study. Data from Garmin smartwatches are routinely synced to Garmin Connect via Ilumivu or Fitabase, which can then be updated for the research study during the patient's weekly visits. Each child's mother or primary caregiver will also be given a Garmin to collect biometric data for exploratory, hypothesis generating data collection. Based on the system for

alerts in both active AI and sham subjects, Bluetooth on the participating parent's phone/device needs to be enabled at all times. Optional blood tests (only if patients and primary caregivers elect to do it) will be collected for exploratory work and ongoing collaborations with the Center for Individualized Medicine. Voice will be recorded for voice analysis and a final Parent Exit Survey will be sent to the parent participant after the completion of therapy. This survey will be distributed via email through REDCap to analyze their experience with the technology.

The current study aims to develop an innovative wearable tracking protocol that will use AI technology. This would be first time such treatment model is used in young children below 8 years old and as such our proposal is innovative in number of ways; it has the potential to begin critical work on innovative assessment strategies for the youngest patients struggling with significant emotional- behavioral issues. Garmin data and artificial intelligence approaches will have enhanced temporal resolution compared to standard assessments. Specifically, these innovations will facilitate: 1) collection of more objective and longitudinal behavioral measures, 2) assessments of standard clinical outcome measures such as the ECBI and PSQ, the rate of tantrum events/duration everyday activities, 3) identify early opportunities for reinforcing positive behavior that are child-specific and 4) understand early signatures of child's behavioral difficulties. Artificial intelligence methods such as reinforcement learning, and probabilistic graphical models will be used to develop methods to analyze data in this project as they are suitable for longitudinal data that can incorporate time-sensitive changes and physician's domain knowledge to make prognoses sensitive and interpretable.

Table 1. Schedule of Study Assessments: Explaining different steps of the study.

Pre-Screening	▪ Identify potential patients from the Young Child Clinic<br>▪ Screening questions that include ECBI
1. Screening, Baseline Assessment and Consent	▪ Consent/Assent<br>▪ Review inclusion and exclusion criteria<br>▪ CBCL, PFC, CCNES, K-SADS, PAPA, C-GAS, ECBI, PSQ, PSI-SF, CGI, TYCYC, ECSA,<br>▪ Demographics, PPH, PMH, FH, SH, medications<br>▪ Biospecimen collection (optional)
2. Post CDI Interval Assessment	▪ CBCL, PFC, CCNES, PSQ, PSI, ECBI
3. Semi-Structured Instruments	▪ All groups: K-SADS, C-GAS, PAPA<br>▪ Participants to be seen by a study child psychiatrist
4. Outcome assessment	▪ CBCL, PFC, CCNES, K-SADS, PAPA, CGI-I, ECBI, PSQ, PSI-SF, TAI, TSCYC<br>▪ Biospecimen collection (optional)<br>▪ Parent Exit Survey

4. Participant Selection, Enrollment and Withdrawal

4.1 Inclusion Criteria

Inclusion Criteria Children:

-Ages 3-7

-Outpatients or Inpatients

-Any gender, race, or ethnicity

-Able to provide developmentally appropriate informed assent, and legal guardians able to provide informed consent

-EBP Severity rated above the clinically significant range (≥120; T-score ≥ 60) (Eyberg Child Behavior Inventory- ECBI; Eyberg & Pincus, 1999).

- Need for more intensive behavioral treatments such as ER visit for behavioral dyscontrol or hospitalization will not be exclusionary or exit criteria.

- Families approached for participation will be asked to commit to complete the treatment

- At least one primary caregiver and the identified child will have to be able to speak and understand English.

Exclusion Criteria Children:

-Formal diagnosis of Severe Intellectual disability, Autistic Spectrum Disorder Level 3, or a psychotic disorder for the child

-Parents not consenting to the study

-Parents or child is not able to adhere to the study protocol

-A Child who is reasonable expected to be unable to tolerate wearing the Garmin device for at least 70% of the time during the day and night 70% of the days during the treatment (12 weeks). This is based on the principal investigator's discretion.

- Unable to speak and understand English

-Refusal or withdrawal of consent, inability, or unwillingness to adhere to study procedures

- Children in foster care

Inclusion Criteria Adults:

- Agree to wear Garmin watch
- Ages 18-99
- Any gender, race, ethnicity

- Able to provide informed consent
- Able to speak and understand English

Exclusion Criteria Adults:

- Unable to speak and understand English.
- Refusal or withdrawal of consent, inability, or unwillingness to adhere to study procedures

4.2 Number of Subjects

50 children and 50 adults (child accompanied by a parent)

4.3 Duration of Participation

12 weeks of PCIT (Parent Child Interaction Treatment) plus one pre and post-evaluation single study visits.

4.4 Participant Recruitment, Enrollment, Screening, Study Procedures and Follow-up

4.4.1 Recruitment

A total of 50 children and 50 legal guardians (randomly assigned to AI-PCIT group- 50 and PCIT- sham biometric group- 50) will be recruited 1:1 (See Figure 3). Patients and their legal guardians will be approached upon evaluation in the Young Child Behavioral Clinic. We will consult patients' primary child psychiatrist to confirm their appropriateness for participation in the study.

Patients of the primary investigator and co-investigators will be offered the opportunity to participate. Participant referrals and peer networking recruiting will be utilized. Other IRB approved studies, research, or biospecimens' databases will be used for recruitment.

Additional resources will be utilized for recruitment of potential participants including:

- from within the clinical and referral practices of the clinical study site
- referrals from other care providers within the treatment communities
- radio advertisements
- invitation letters sent to parents of potentially eligible participants
- print advertisements, including but not limited to brochures, flyers, and listings in research classifieds
- study listing on university and clinical study site website
- social media

All advertising materials will be reviewed and approved by the Mayo Clinic IRB prior to use.

Potentially interested families may be provided with printed participant educational materials to aid them in making an informed decision regarding their willingness to participate in this study.

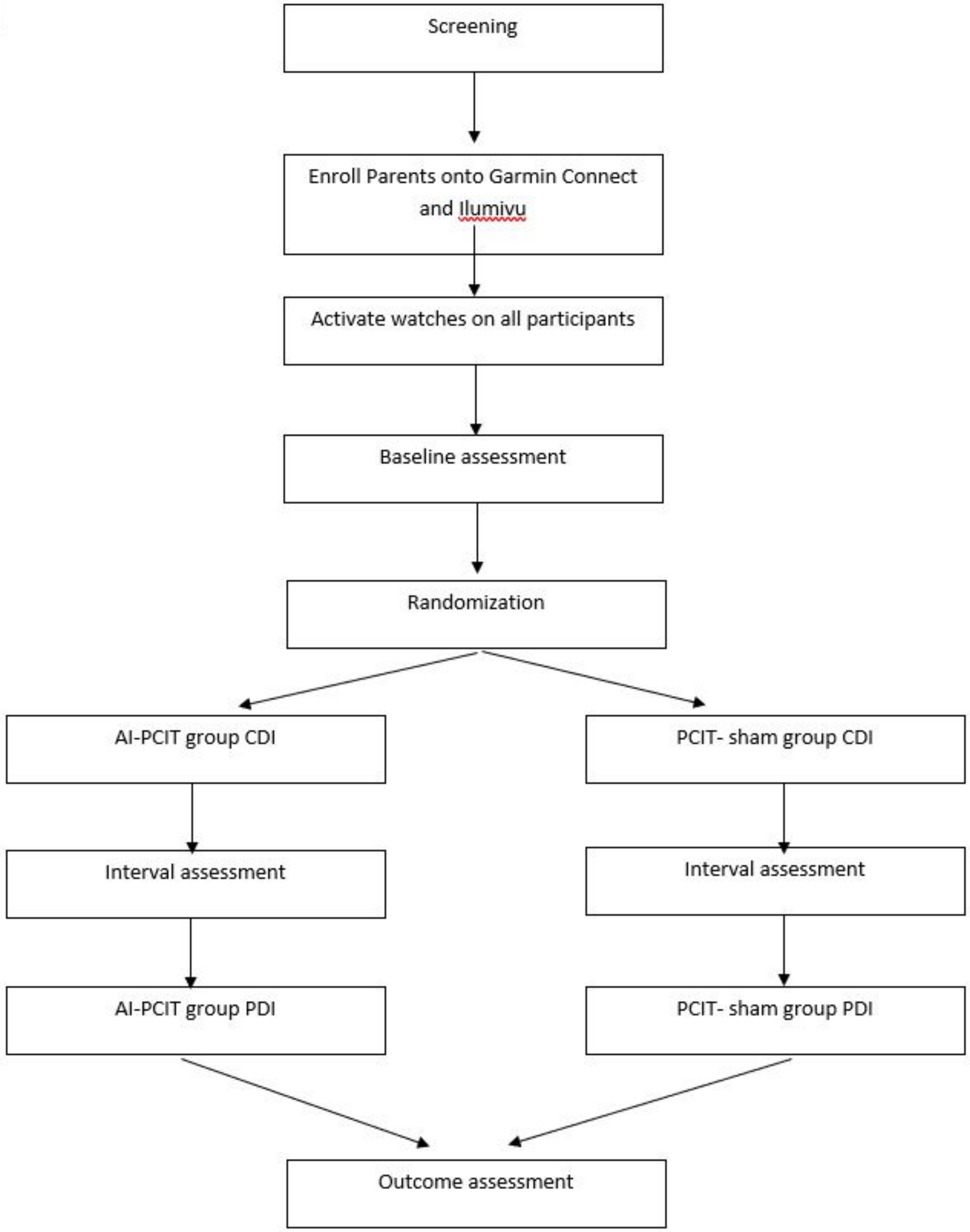

Figure 3. Study Schema

4.4.2 Pre-screening

Pre-screening will be carried out over the phone or in person for all interested legal guardians of children 3-7 yo who meet inclusion criteria. Screening will include study overview and discussing information related to inclusion and exclusion criteria. Those who are deemed eligible will be scheduled for the study visit.

4.4.3 Consent/Assent

Institutional Review Board (IRB) approval will be obtained for the consent and assent form. The study staff will describe the study in full, including the risks and benefits of the study. After ample time for questions and verifying understanding of the study, the participant and parent will then be asked to sign the consent and assent forms prior to participation. Electronic consent will be an option. Dr. Romanowicz or a research team member that Dr. Romanowicz has trained and supervises, will explain the details of the study, answer any study related questions, and obtain informed assent and consent from the participants and their parents or legal guardians. Dr. Romanowicz or a board-certified child and adolescent psychiatrist will be available to address any questions participants may have that the person obtaining consent cannot answer and those that arise throughout the study. Dr. Romanowicz and the study team will reinforce that declining study participation does not impact accessibility to standard psychiatric treatment within the Mayo Clinic system or surrounding community. Dr. Romanowicz will offer ongoing standard clinical care or appropriate referrals (based on patient preference) to patients who exit the study or complete all study procedures.

4.4.4 Screening

After providing informed consent and assent, participants will undergo an initial assessment for screening with questionnaires for demographic data, CBCL (Child Behavior Checklist), PFC (Preschool Feelings Checklist), CCNES (Coping with Children's Negative Emotions), K-SADS (Kiddie Schedule for Affective Disorders and Schizophrenia), PAPA (Preschool Age Psychiatric Assessment), C-GAS (Children Global Assessment Scale), ECBI (Eyberg Child Behavior Inventory), PSQ (Pediatric Sleep Questionnaire, PSI-SF (Parenting Stress Index-Short Form), CGI-I (Clinical Global Impression – Global Improvement), TSCYC (Trauma Symptom Checklist for Young Children), ECSA (Early Childhood Screening Assessment), psychiatric history, medical history, family history, social history, and medication history.

The study participants will be offered the opportunity to contribute biospecimens for the exploratory aspects of the study (blood). If indicated, study participants will undergo venipuncture for the collection of CRP, genomewide genomics, proteomics, exposomics, and metabolomics. If study participants choose to participate in the CRP collection, results of the specimen would be made available to the research participant via portal message.

4.4.5 Study Procedures

Following screening, participants will be randomly assigned to AI-PCIT group and PCIT- sham biometric group. The child and primary caretaker will be fitted with Garmin watches that they will be required to wear at least during 70% of day and night each day for 70% of days during 12 weeks of the treatment. Families will be scheduled for PCIT treatment with PCIT certified therapist in Young Child Behavioral Clinic. Parents in the AI- PCIT group are going to receive targeted messages during tantrums picked up by Garmin devices that will instruct them on how to deescalate their child. Parents in the PCIT-sham group will receive random messages throughout the day with various relaxation strategies such as reminders to practice deep breathing with their child. The treatment will be overseen by Dr. Romanowicz who is a Within Agency PCIT trainer and board-certified child and adolescent psychiatrist.

Dr. Romanowicz (or a board-certified child and adolescent psychiatrist designee) will assess all research participants (AI-PCIT and PCIT- sham biometric) at the conclusion of the study. Any relevant clinical or research findings will be immediately communicated to the clinical treatment team. If patient is deemed to require continuation of treatment at the conclusion of the study, they will be referred for standard of care treatment.

4.5 Early Withdrawal of Participants

4.5.1 Participant Withdrawal, Data Collection, and Follow-Up

Participants may withdraw voluntarily from the study at any time. Participants will be withdrawn from the study by the PI/Sponsor if a participant:

- is non-compliant with study procedures
- Meets exclusion criteria at any point after consent

The PI may also withdraw a participant if he/she believes that for safety reasons it is in the best interest of the participant to be withdrawn.

Discontinuation information [e.g., date and the reason(s) for discontinuation] will be recorded in the Participant's CRF (Case Report Form).

Participants withdrawn from the study due to an adverse event will be followed up for 30 days or until resolution. Participants withdraw from the study will not be replaced, regardless of the reason for withdrawal. An effort will be made to determine why a participant does not return for the required visits or is dropped from the study. This information will subsequently be recorded on the participant's CRF.

Participants will be encouraged to remain compliant with all expected study procedures and visits. Non-adherence to expected study procedures will be documented and may result in removal from the study. This will be clearly discussed during the consent/assent process and reinforced throughout the study through regular screening for issues with compliance.

Dr. Romanowicz will assist with clinical referral for all participants who voluntarily withdraw from the study or are withdrawn by the research team and PI. Specifically, Dr. Romanowicz will offer mental health referrals based on standard of care and the preference of the participant. Participants will also be advised of useful Mayo Clinic Patient Education Materials.

5. Study Procedures, devices, and instruments

5.1 Pre-screening

Pre-screening will be carried out over the phone or in person for all interested participants and their legal guardians. Screening will include study overview and discussing information related to inclusion and exclusion criteria. Those who are deemed likely to be eligible will be scheduled for the study screening visit.

5.2 Study Visit 1

5.2.1 Part 1- Screening

1) Review of Inclusion and exclusion criteria: A team member will review the inclusion and exclusion criteria with the participant and their legal guardian or parent.

2) Consent and Assent: Informed assent and consent will be obtained from the participants and their parents or legal guardians. After providing informed consent and assent, following data will be collected:

3) Demographics and Personal History

Psychiatric history: Psychiatric history will include past psychiatric diagnosis, treatments, therapy history, hospitalizations.

Medical history: Any past or current medical diagnoses and treatments.

Family history: Family history of psychiatric diseases, treatments, suicide attempts or completed suicides, medical history

Social history: Developmental history, living situations, socioeconomic status, family structure, and education. Abuse history and adverse life event will be obtained by structured instruments in Part 3 using Trauma Symptom Checklist for Young Children.

Medication history: List of current and previous medications, dosages, and duration of use.

5) Diagnostic Interviews

1) **K-SADS-EC** is a semi-structured clinical interview for DSM-5 disorders adapted for use in children aged 3.0-6. This measure has test re-test reliability and construct validity that generates both categorical and dimensional measures of DSM-5 Axis I disorders.

2) **The PAPA** is a Parent-Report only interviewer-based structured diagnostic interview. Derived from the Child and Adolescent Psychiatric Assessment (CAPA), it is tailored to feelings and behaviors pertinent to young children. The PAPA was originally developed for use with preschoolers aged 2 to 5 years old. However, it is widely used with 2 to 8 year old children for the diagnosis of the full range of common psychiatric disorders.

5.2.2 C - reactive protein (CRP)

Participants (both parent and child) will undergo an optional venipuncture for CRP collection at baseline and post-treatment (after 12 weeks). If indicated, the draw will be completed within 14 days after providing consent to the study for baseline and within 14 days before their final assessment for post treatment. C - reactive protein is a lab test that is used to assess the presence of inflammation. Prior work has demonstrated that CRP may have utility in guiding the selection of psychiatric treatments. A total of 11mL of blood will be drawn from the participant for the initial draw. Of the total, 5mL, collected in a red top serum tube, will be used to run the CRP lab test at Mayo Clinic. A total of 5mL of blood will be drawn from the participant for the final draw, all of which will be collected in a red top serum tube for CRP testing. If the full volume of blood is not able to be obtained, lab minimums will be used instead.

5.2.3 Plasma Multi-Omics

An exploratory aim that will optionally collect plasma from both the child and the participating parent to characterize multi-omic associations of disruptive behaviors in a parent-child dyad study and associations with physiological smartwatch measures. If indicated, the draw will be completed within 14 days after providing consent to the study and is only tested for once. Four different omics will be tested at the end of the study: metabolomics, exposomics, proteomics, and genomics. Of the initial 11mL draw, 6mL will be collected in a purple top EDTA tube. The sample will be processed and aliquoted into 6 x 0.5mL of plasma and 1 x WBC for DNA extraction. These samples will be stored at -80C until the end of the study when they will be shipped to their respective facilities for analysis. One 0.5mL aliquot of plasma will be sent and analyzed for proteomics through OLINK at their AS-Waltham lab. Two 0.5mL aliquots of plasma will be used for metabolomics and exposomics and will be analyzed at Emory. The 1 x WBC aliquot will be used for DNA extraction. The genetic DNA will be plated with a minimum of 250ng for analysis at Mayo Clinic. If the full volume of blood is not able to be obtained, lab minimums will be used instead. All remaining aliquots will be stored at -80C for future analysis should it be indicated on the consent form.

5.2.4 Part 2- Smartwatch measures

We propose to use Garmin Vivosmart4 trackers (previously used in IRB application 20-002133 preliminary study) to monitor the step count, sleep quality, heart rate (resting and active) and activity rates (i.e., how often subject moves). These data are available with granularity of minutes and will be recorded through a web-interface hosted by Garmin. All participants will be fitted with a Garmin watch after they consent to the study. Study staff will walk the parents/guardians through on how to download a Garmin application on their phone that will allow for confidential syncing of the data from the watch to a web-interface hosted by Garmin on a weekly basis, ideally at the time of their PCIT appointment. Garmin watches are waterproof,

and participants will be encouraged to wear them at all times during the 12 weeks of treatment (minimum of 70% of the time, 70% of the days).

5.2.5 Part 3- Psychiatric Interview and Questionnaires

-*The Kiddie Schedule for Affective Disorders and Schizophrenia-Early Childhood Version (K-SADS-EC)* is a semi-structured clinical interview for DSM-5 disorders adapted for use in children aged 3.0-6. This measure has test re-test reliability and construct validity that generates both categorical and dimensional measures of DSM-5 Axis I disorders.

-*The Preschool Age Psychiatric Assessment (PAPA)* is a Parent-Report only interviewer-based structured diagnostic interview. Derived from the Child and Adolescent Psychiatric Assessment (CAPA), it is tailored to feelings and behaviors pertinent to young children. The PAPA was originally developed for use with preschoolers aged 2 to 5 years old. However, it is widely used with 2 to 8-year-old children for the diagnosis of the full range of common psychiatric disorders.

-*Children's Global Assessment Scale (CGAS)* The CGAS is a standardized instrument that measures children's global level of impairment completed by the clinician-rater.

-*Clinical GlobaI Impression – Global Improvement (CGI-I)* The CGI-I is a 7-point Likert scale widely used in treatment research that measures the blind clinician-rater's impression of improvement at post assessment.

-*Coping with Children's Negative Emotions (CCNES)* The CCNES is a valid and reliable caregiver report measure of parental coping styles and strategies in response to children's expression of negative emotions.

-*The Eyberg Child Behavior Inventory* (ECBI) is a 36-item parent-report measure of the frequency of their 2-16-year-old child's disruptive behaviors. The ECBI is a 36-item questionnaire that helps to assess the frequency of externalizing behaviors in children (2-16 years old).

-*The Parenting Stress Index-Short Form (PSI-SF)* is a 36-item measure of parental stress. The PSI will be administered at baseline, 2 weeks, and 12 weeks and completed by the primary caregiver.

-*The Child Behavior Checklist (CBCL)* is a checklist parents complete to detect emotional and behavioral problems in children and adolescents.

-*The Pediatric Sleep Questionnaire (PSQ)* is a retrospective, 22-item parent questionnaire used to examine sleep behavior in young children.

-*The Preschool Feelings Checklist* (PFC) is a 20-item parent report checklist designed to identify preschoolers (ages 3.0–5.6 years) with symptoms of depression. The PFC is a highly feasible yes/no checklist that takes only 2 to 4 minutes to complete by a parent or caregiver about his or her preschool child.

-*The Therapy Attitude Inventory* (TAI) Treatment Satisfaction will be measured at the end of the treatment for AI-PCIT group and PCIT sham at the end of the treatment. For those families who

do not complete 2 weeks attempts will be made to complete the TAI over the phone at point of last contact.

- *Trauma Symptom Checklist for Young Children* (TSCYC) is a 90-item caretaker-report instrument developed for the assessment of trauma-related symptoms in children ages 3-12. It contains two reporter validity scales and eight clinical scales. The scales allow a detailed evaluation of posttraumatic stress symptoms and a tentative PTSD diagnosis. It also provides information on other symptoms such as anxiety, depression, anger, and abnormal sexual behavior.

- *Early childhood screening assessment (ECSA)* A primary care screening measure developed to identify very young children (1½-5 years old) who need further emotional or behavioral assessment. The ECSA was developed specifically to meet the logistical constraints of primary care settings.

- *Dyadic Parent-Child Interaction Coding System II (DPICS-II)* A coding system for specific structured parent-child interactions targeted by PCIT. DPICS-II includes codes for parent behaviors (e.g., commands, criticism, labeled praise, etc.) and child behaviors (e.g., noncompliance, compliance, etc.). Commonly given clinically and will be using the results for research. If not given clinically then will be performed for research purposes only.

- Behavioral Measures from Wearable Technologies: We propose to use Garmin trackers to monitor the step count, sleep quality, heart rate (resting and active) and activity rates (i.e., how often subject moves). These data are available with granularity of minutes and will be recorded through a web-interface hosted by Garmin.

- PISTACHIo Parent/Guardian Exit Interview: This questionnaire is distributed to learn more about parent's experience with the technology used in the study.

5.3 Post CDI Interval Assessment Visit

After 6 CDI sessions participants from both arms of the study AI-PCIT and PCIT- sham biometric will undergo:

- Review of Inclusion and exclusion criteria
- Review of interim psychiatric, medical and social history
- Review of interim medical and medication history
- Psychiatric Interview and Questionnaires
    1) CBCL (Child Behavior Checklist)
    2) PFC (Preschool Feelings Checklist)
    3) CCNES (Coping with Children's Negative Emotions)
    4) PSQ (Pediatric Sleep Questionnaire)
    5) PSI (Parenting Stress Index)
    6) ECBI (Eyberg Child Behavior Inventory)

If participants are unwilling to return for the follow-up visit, they will be asked to answer some of questionnaires over the phone.

5.4 Outcome Assessment Visit

At the end of the treatment participants from both arms of the study AI-PCIT and PCIT- sham biometric will undergo:

- Review of interim psychiatric, medical, and social history
- Review of interim medical and medication history
- Venipuncture for posttreatment CRP
- Psychiatric Interview and Questionnaires

1) CBCL (Child Behavior Checklist)
2) PFC (Preschool Feelings Checklist)
3) CCNES (Coping with Children's Negative Emotions)
4) K-SADS-EC (Kiddie Schedule for Affective Disorders and Schizophrenia-Early Childhood)
5) PAPA (Preschool Age Psychiatric Assessment)
6) CGI-I (Clinical Global Impression – Global Improvement)
7) ECBI (The Eyberg Child Behavior Inventory)
8) PSQ (Pediatric Sleep Questionnaire)
9) PSI-SF (Parenting Stress Index-Short Form)
10) TAI (The Therapy Attitude Inventory)
11) TSCYC (Trauma Symptom Checklist for Young Children)
12) PISTACHIo Parent/Guardian Exit Interview

5.5 *PCIT Treatment*

PCIT is going to be implemented in two stages: Child Directed Interaction (CDI) and Parent Directed Interaction (PDI). CDI will involve teaching PRIDE (Praise, Reflection, Imitation, Description, and Enthusiasm) "do" skills that the caregiver is going to be taught to utilize during interactions and play with the child. At the same time, the caregiver is going to be taught to avoid "don't" skills during CDI. Once mastery of the PRIDE skills will be attained (typically session 6), the therapy will transition onto PDI. Here, the caregiver will learn appropriate disciplinary techniques, such as effective time-outs, and the phrasing of clear directions or commands (direct commands). Sessions are usually 45 min long, although in the second part of treatment- PDI, they may be extended to 60–90 min depending on the child's response. For example, if the child does not comply with a direct command given to them by their parent, they will have to sit in a time out chair for 3 min followed by 5 seconds of silence (the latter is important for the child to learn that only calm behavior will get them out of the chair). Likewise, if the child gets off the chair prematurely, they must go to the time out room. Parents will be asked to wait at least a minute that is followed by 5 seconds of silence before approaching the child to put them in the time out chair. PDI will also include a public outing session where parents will have the opportunity to practice their newly acquired skills in a hospital store. There will also be a sibling session (if applicable) where parents will practice their skills with all their children.

All sessions are going to be conducted by PCIT certified therapists overseen by Dr. Romanowicz who is PCIT within agency trainer. The 2011 PCIT Protocol (Eyberg & Funderburk, 2011) (18) will be followed to conduct the sessions.

5.6 *Biomarker S*ample *C*ollections

COLLECTIONS

Blood collection

Peripheral blood specimens (no more than 3 mL/Kg per lab draw will be taken to comply with typical minimal risk standard) will be collected on-site at one of the outpatient phlebotomy stations at Mayo Clinic. The collected specimens will be stored in -80C degree freezers. 11mL of blood will be collected from all subjects who opt into this choice. Blood will be collected for CRP testing and multi-omic analysis.

If indicated on the consent form, these samples will continue to be stored for future additional assays of untargeted metabolomics and environmental exposures.

Voice analysis

All sessions are going to be recorded (audio and video). Child's scream voice recording is going to be used to voice analysis as a reflexion of cardiac vagal nerve regulation.

Table 1. Description of Assessments

Assessment	Description
Adverse events	Participants will be evaluated for adverse events at each visit. Serious adverse events (SAEs) will be reported as they occur to the Mayo Clinic IRB and USA FDA (as per regulations).
Kiddie-Schedule for Affective Disorders and Schizophrenia-Early Childhood (K-SADS-EC)	Semi-structured clinical interview for DSM-5 disorders adapted for use in children aged 3.0-6. This measure has test re-test reliability and construct validity that generates both categorical and dimensional measures of DSM-5 Axis I disorders.
Preschool Age Psychiatric Assessment (PAPA)	Parent-Report only interviewer-based structured diagnostic interview. Derived from the Child and Adolescent Psychiatric Assessment (CAPA), it is tailored to feelings and behaviors pertinent to young children. The PAPA was originally developed for use with preschoolers aged 2 to 5 years old. However, it is widely used with 2 to 8-year-old children for the diagnosis of the full range of common psychiatric disorders.

Assessment	Description
Children's Global Assessment Scale (CGAS)	The CGAS is a standardized instrument that measures children's global level of impairment completed by the clinician-rater.
Clinical GlobaI Impression – Global Improvement (CGI-I)	The CGI-I is a 7-point Likert scale widely used in treatment research that measures the blind clinician-rater's impression of improvement at post assessment.
Coping with Children's Negative Emotions (CCNES)	The CCNES is a valid and reliable caregiver report measure of parental coping styles and strategies in response to children's expression of negative emotions.
The Eyberg Child Behavior Inventory (*ECBI*)	36-item parent-report measure of the frequency of their 2-16-year-old child's disruptive behaviors. The ECBI is a 36-item questionnaire that helps to assess the frequency of externalizing behaviors in children (2-16 years old).
Parenting Stress Index-Short Form (PSI-SF)	36-item measure of parental stress. The PSI will be administered at baseline, 2 weeks, and 12 weeks and completed by the primary caregiver.
CBCL- The Child Behavior Checklist	Parent report form to screen for emotional, behavioral, and social problems in eight different categories providing a quantitative measure of internalizing and externalizing symptoms.
The Pediatric Sleep Questionnaire (PSQ)	A retrospective, 22-item parent questionnaire used to examine sleep behavior in young children.
The Preschool Feelings Checklist (*PFC*)	20-item parent report checklist designed to identify preschoolers (ages 3.0–5.6 years) with symptoms of depression. The PFC is a highly feasible yes/no checklist that takes only 2 to 4 minutes to complete by a parent or caregiver about his or her preschool child.
Behavioral Measures from Wearable Technologies	We propose to use Garmin trackers to monitor the step count, sleep quality, heart rate (resting and active) and activity rates (i.e., how often subject moves). These data are available with granularity of minutes and will be recorded through a web-interface hosted by Garmin.
The Therapy Attitude Inventory (*TAI*)	Treatment Satisfaction will be measured at the end of the treatment for AI-PCIT group and PCIT sham at the end of the treatment. For those families who do not complete 2 weeks attempts will be made to complete the TAI over the phone at point of last contact.
Trauma Symptom Checklist for Young Children (*TSCYC*)	90-item caretaker-report instrument developed for the assessment of trauma-related symptoms in children ages 3-12. It contains two reporter validity scales and eight clinical scales. The scales allow a detailed evaluation of posttraumatic stress symptoms and a tentative PTSD diagnosis. It also provides information on other symptoms such as anxiety, depression, anger, and abnormal sexual behavior.

Assessment	Description
Early childhood screening assessment (ECSA)	Primary care screening measure developed to identify very young children (1½-5 years old) who need further emotional or behavioral assessment. The ECSA was developed specifically to meet the logistical constraints of primary care settings.
C-Reactive Protein (CRP)	CRP is a lab test that is used to assess the presence of inflammation. Prior work has demonstrated that CRP may have utility in guiding the selection of psychiatric treatments.
Multi-omics	An exploratory aim that will collect plasma from both the child and the participating parent to characterize multi-omic associations of disruptive behaviors in a parent-child dyad study and with physiological measures from smartwatches.
Dyadic Parent-Child Interaction Coding System II (DPICS-II)	A coding system for specific structured parent-child interactions targeted by PCIT. DPICS-II includes codes for parent behaviors (e.g., commands, criticism, labeled praise, etc.) and child behaviors (e.g., noncompliance, compliance, etc.). Commonly given clinically and will be using the results for research. If not given clinically then will be performed for research purposes only.
PISTACHIo Parent/Guardian Exit Interview	A brief questionnaire to help understand the parent participant's experience with technology used in the study. This information will help refine and develop use of technology for future studies.

Table 2. Timeline of Assessments

	Pre-screening	Screening/Baseline Assessment	Daily	Weekly	CDI sessions (6)	Interval Assessment	PDI sessions (6)	Outcome Assessment
**Brief review of inclusion and exclusion criteria**	**X**							
Informed Consent/Assent/ HIPAA		**X**						

Review of inclusion and exclusion criteria		**X**						
PAPA		**X**						**X**
K-SADS		**X**						**X**
Family History		**X**				**X**		
Social History		**X**				**X**		
Past Medical/Psychiatric History		**X**						
Medications						**X**		
**Questionnaires**								
CBCL		**X**				**X**		**X**
PFC		**X**				**X**		**X**
CCNES		**X**				**X**		**X**
C-GAS		**X**						
PSQ		**X**		**X**	**X**	**X**	**X**	**X**
PSI-SF		**X**				**X**		**X**
CGI-I		**X**						**X**
ECBI		**X**		**X**	**X**	**X**	**X**	**X**
TAI								**X**
TSCYC		**X**						**X**
ECSA		**X**						
Venipuncture for CRP		**X**						**X**
Genomic Analysis		**X**						
Parent Exit Interview								**X**
**Garmin**			**X**					
**Illumivu**			**X**					

Table 2. PPH: psychiatric history, MH: medical history, FH: family history, SH: social history, CBCL: Child Behavior Checklist; CCNES: Coping with Children's Negative Emotions; CDI: Child Directed Intervention; PDI-Parent Directed Interaction; C-GAS: Children Global Assessment Scale: K-SADS: Kiddie Schedule for Affective Disorders and Schizophrenia; PFC: The Preschool Feelings Checklist, PAPA: Preschool Age Psychiatric Assessment; CGI-I: Clinical Global Impression – Global Improvement; ECBI: The Eyberg Child Behavior Inventory; PSQ: Pediatric Sleep Questionnaire; PSI-SF: Parenting Stress Index-Short Form (PSI-SF); TAI: The Therapy Attitude Inventory; TSCYC: Trauma Symptom Checklist for Young Children; ECSA- Early Childhood Screening Test;

6. Statistical Plan

6.1 Study purpose

The primary goal of the study is to establish feasibility of the proposed augmentations to PCIT. This study is an exploratory clinical trial with the broad goals of demonstrating feasibility, generating pilot data, and to generate validate effect sizes, to inform future larger, multicenter trials.

6.2 *Statistical Methods*

All statistical analyses will be carried out using SAS software, version 9.4 or STATA MP 14. The level of significance will be set at $\alpha = .05$ (two-tailed) and, to address multiple testing (where applicable), p-values will be adjusted using the False Discovery Rate.

6.3 *Sample Size Determination*

Sample Size Estimation and Power Analysis for the Primary Aim of the Study

We estimated the sample size for this pilot study based on the hypothesis examining clinical outcome (ECBI) between the PCIT-AI group and PCIT sham group (hypothesis 2a). We anticipate that a sample size of 50 participants per group (N=100) achieves 70% power, at a 0.05 alpha level (two-tailed) to detect an odds ratio of 2.0 favoring PCIT-AI with the proposed randomized design. This estimation was based on prior work and knowledge of PICT within the context of an exploratory trial. The sample size is designed in part to detect a moderate effect and establish a proof of concept to inform a larger, multicenter trial.

6.4 Data Analysis Plan for Aim 1

Hypothesis 1 will be examined with descriptive statistics assessing the frequency Garmin use in participants (70% or greater is defined as success for this hypothesis).

6.5 *Data Analysis Plan for Aim 2*

Hypotheses 2a and 2b focuses on a primary continuous outcome measure (ECBI) over 12 weeks. A linear mixed model analysis of repeated measures will be used to evaluate the interaction effects of type of PCIT (AI informed or AI sham), time, and ECBI scores. A separate linear

mixed model analysis will be used to evaluate the interaction effects of type of PCIT (AI informed or AI sham), time, and PSQ scores with ECBI scores as a covariate.

6.6 Data Analysis Plan for Aim 3 and Exploratory Aims

Hypotheses 3a and 3b will be examined with Spearman correlation coefficients and mixed linear regression models to examine the relationships among biometric data from Garmin watches (sleep data) and the PSQ. Separate Spearman correlations and a mixed linear regression model will examine the relationship between biometric data from Garmin watches (heart rate and movement) with weekly ECBI reports.

Partial least-sqaure regression networks will be used to derive multi-omic integration networks and Spearman correlation will then be used to associate omics with physiological measures from smartwatches.

7. Treatment and Safety Plan

7.1 Treatment

This study includes standard evidenced based treatment for oppositional behaviors and emotional behavioral disorders in children 3- 7 years old: Parent Child Interaction Therapy. Treatment is going to be provided by certified therapist overseen by Dr. Romanowicz who is a within agency PCIT trainer.

PCIT is an evidence-based behavioral treatment for 3–7 year olds with behavioral disturbances arising from internalizing and externalizing disorders. The aim of treatment is to improve symptoms by improving the child–caregiver relationship. The distinguishing feature of PCIT is the use of a “bug in the ear system” that allows the therapist to coach the caregiver in real time. This discrete method allows for in the moment training and feedback as the caregiver interacts with their child while the therapist watches and teaches behind a one-way mirror.

PCIT is implemented in two stages: Child Directed Interaction (CDI) and Parent Directed Interaction (PDI). CDI involves teaching PRIDE (Praise, Reflection, Imitation, Description, and Enthusiasm) “do” skills that the caregiver is taught to utilize during interactions and play with the child. At the same time, the caregiver is taught to avoid “don’t” skills during CDI. Once mastery of the PRIDE skills is attained, the therapy transitions onto PDI. Here, the caregiver learns appropriate disciplinary techniques, such as effective time-outs, and the phrasing of clear directions or commands (direct commands). Sessions are usually 45 min long, although in the second part of treatment- PDI, they may be extended to 60–90 min depending on the child’s response. For example, if the child does not comply with a direct command given to them by their parent, they have to sit in a time out chair for 3 min followed by 5 seconds of silence (the latter is important for the child to learn that only calm behavior will get them out of the chair). Likewise, if the child gets off the chair prematurely, they have to go to the time out room, or if the room is unavailable, parents practice a “swoop and go” strategy, where they collect all the

toys that are in the room and tell the child that they will be standing right outside the door until the child is ready to sit in the chair. The recommendation is that parents should wait at least a minute that is followed by 5 seconds of silence before approaching the child to put them in the time out chair. PDI also includes a public outing session where parents have the opportunity to practice their newly acquired skills in a park or a store. There is also a sibling session where parents practice their skills with all their children. This is particularly helpful for children who have difficult relationships with their siblings.

PCIT has a wide body of evidence supporting its use, including randomized controlled trials indicating long term improvement in parenting strategies and diminished behavioral issues, as compared with treatment-as-usual groups. Based on comparative studies, PCIT demonstrated large effect sizes for helping with negative parent and child behaviors as well as increasing positive parenting skills and improving child behaviors. There are at least 12 meta-analyses and reviews of PCIT research. Based on one analysis, PCIT has a large effect size of d = 1.65 (decrease in externalizing symptoms in children).

7.2 Safety Plan

No treatment is funded during the course of this study. The participants will be recruited from the Young Child Behavioral Clinic (YCBC) and will be initiating PCIT treatment that is billed clinically. For the patient group we will consult with the patients' primary team to assess patients' appropriateness for participating in the study. Dr. Romanowicz is a board-certified child and adolescent psychiatrist with extensive clinical experience in treatment of young children 3-7. All patients in our study will receive the standard evidence-based treatment Parent Child Interaction Therapy (PCIT) during the course of this study. They will also be allowed to receive medications if their primary care clinician deems it appropriate.

Note that in the course of the study evaluation and clinical measures the PI (Dr. Croarkin) and study psychiatrists will take all necessary steps to keep study participants safe as required by law and within the standard of care. For example, this could involve contacting county or state child protection services for a minor who has had prior or ongoing abuse or neglect. All clinical measures and rating scales will be reviewed during the study visit by the PI (Dr. Romanowicz) or a PhD or MD level co-investigator.

7.3 *Data and Safety Monitoring*

This study will be monitored by the Investigator according to the monitoring plan described below. The Data and Safety Monitoring Plan will be in place to ensure the safety of the study, the study patients, and the scientific validity and integrity of data collected as part of the study.

7.3.1 Subject Safety

a) Safety will be monitored by the Investigator and study team.

b) Participants will be seen by Dr. Romanowicz or another board-certified child psychiatrist at every study visit and the evaluation will be documented.
c) Participants will be monitored for adverse events (AEs) throughout every visit.

7.3.2 Subject Removal from Study

a) Subjects may be removed from study participation for any of the following reasons:
    a. Failure to meet inclusion/exclusion criteria following pre-screening
    b. Withdrawal of consent
    c. Lost to follow-up
    d. Other reasons, such as specified administrative reasons
b) The Principal Investigator will make the final decision about termination of study participation

7.3.3 Reporting Mechanisms

a) IRB Reports
    a. Investigator will determine if event is an Unanticipated Problems Involving Risk to Participants (UPIRTSO) or Others or non-UPIRTSO
    b. UPIRTSOs must be reported to the IRB within 5 working days of knowledge of problem or event (per IRB procedure)
    c. If Investigator determines event to be a non-UPIRTSO, event will be reported at continuing review

7.3.4 Data Integrity

a) Data is entered into REDCap and preserved in read-only files
b) Study data will be reviewed regularly by the Investigator and Study Coordinator for the following:
    a. Subject inclusion criteria have been met
    b. Transcription of data is accurate and complete
    c. Units of measure are recorded appropriately

7.3.5 Subject Privacy

a) Study visits will take place in a confidential setting

7.3.6 Data Confidentiality

a) Non-electronic source document data will be stored in a locked cabinet in a secure office. Only authorized study staff will have access.
b) Electronic data will be stored on a secure database. Only authorized users will have access. All users will have unique identifiers and passwords. Sharing of log-in information is not permitted.

7.3.7 Product Accountability

a) Not applicable

7.3.8 Study Documentation

a) Quality assurance will be conducted quarterly by the study team to assure that required documentation and reports are on file, accurate, and completed

7.3.9 Study Coordination

a) Study staff will be thoroughly educated about the protocol and requirements of the study
b) All study activities will be conducted by study staff within their appropriate scope of license and education
c) Study staff will have regular meetings to ensure that the study is conducted in a systematic manner by all who contribute
d) Study team will have open communication to assure that ideas and concerns are addressed in a timely manner

8. Data Handling and Record Keeping

8.1 Confidentiality

Information about study Participants will be kept confidential and managed according to the requirements of the Health Insurance Portability and Accountability Act of 1996 (HIPAA). Those regulations require a signed Participant authorization informing the Participant of the following:

- What protected health information (PHI) will be collected from Participants in this study
- Who will have access to that information and why
- Who will use or disclose that information
- The rights of a research Participant to revoke their authorization for use of their PHI.

In the event that a Participant revokes authorization to collect or use PHI, the investigator, by regulation, retains the ability to use all information collected prior to the revocation of Participant authorization. For Participants that have revoked authorization to collect or use PHI, attempts should be made to obtain permission to collect at least vital status (long term survival status that the Participant is alive) at the end of their scheduled study period.

8.2 Source Documents

Source data comprise all information, original records of clinical findings, observations, or other activities in a clinical study necessary for the reconstruction and evaluation of the study. Source data are contained in source documents. Examples of these original documents, and data records include: hospital records, clinical and office charts, laboratory notes, memoranda, participants' diaries or evaluation checklists, pharmacy dispensing records, recorded data from automated instruments, copies or transcriptions certified after verification as being accurate and complete, microfiches, photographic negatives, microfilm or magnetic media, x-rays, participant files, and

records kept at the pharmacy, at the laboratories, and at medico-technical departments involved in the study. When applicable, information recorded on the CRF shall match the Source Data recorded on the Source Documents.

9. Study Finances

9.1 Funding Source

Mayo Foundation.

9.2 Participant Stipends or Payments

We will provide reimbursement for participation in the study as long as the participant has a Mayo Clinic Number. The parent participant will receive $50 per week so long as the dyad completes both the weekly therapy session and surveys. Each participant (parent and child) will be paid $25 per optional blood draw.

Publication Plan

A publication committee will be formed and co-chaired by Drs. Romanowicz and Athreya. This committee will hold the primary responsibility for publications of results from the proposed study.

10. References

1. Brown CM, Copeland KA, Sucharew H, Kahn RS. Social-emotional problems in preschool-aged children: opportunities for prevention and early intervention. Arch Pediatr Adolesc Med. 2012;166(10):926-32.
2. Nolan EE, Gadow KD, Sprafkin J. Teacher reports of DSM-IV ADHD, ODD, and CD symptoms in schoolchildren. J Am Acad Child Adolesc Psychiatry. 2001;40(2):241-9.
3. Eyberg SM, & Pincus, D. Eyberg Child Behavior Inventory and Sutter-Eyberg Student Behavior Inventory-Revised: Professional Manual. . Odessa, FL: Psychological Assessment Resources 1999.
4. Upshur C, Wenz-Gross, M., & Reed, G. . A pilot study of early childhood mental health consultation for children with behavioral problems in preschool. Early Childhood Research Quarterly. 2009;24(1), 29-45.
5. Kupersmidt JB, Bryant, D., & Willoughby, M. T. . Prevalence of aggressive behaviors among preschoolers in Head Start and community child care programs. Behavioral Disorders. 2000;26(1), 42-52.
6. Loeber R, Farrington DP. Young children who commit crime: epidemiology, developmental origins, risk factors, early interventions, and policy implications. Dev Psychopathol. 2000;12(4):737-62.
7. Cuda SE, Censani M. Pediatric Obesity Algorithm: A Practical Approach to Obesity Diagnosis and Management. Front Pediatr. 2018;6:431.
8. Clearinghouse for Child Welfare (http://www.cebc4cw.org)- accessed on 7/7/2021.

9. Thomas R, Abell B, Webb HJ, Avdagic E, Zimmer-Gembeck MJ. Parent-Child Interaction Therapy: A Meta-analysis. Pediatrics. 2017;140(3).
10. Lanier P, Kohl PL, Benz J, Swinger D, Moussette P, Drake B. Parent-Child Interaction Therapy in a Community Setting: Examining Outcomes, Attrition, and Treatment Setting. Res Soc Work Pract. 2011;1(6):689-98.
11. Comer JS, Furr JM, Miguel EM, Cooper-Vince CE, Carpenter AL, Elkins RM, et al. Remotely delivering real-time parent training to the home: An initial randomized trial of Internet-delivered parent-child interaction therapy (I-PCIT). J Consult Clin Psychol. 2017;85(9):909-17.
12. Runkle J, Sugg M, Boase D, Galvin SL, C CC. Use of wearable sensors for pregnancy health and environmental monitoring: Descriptive findings from the perspective of patients and providers. Digit Health. 2019;5:2055207619828220.
13. Heale LD, Dover S, Goh YI, Maksymiuk VA, Wells GD, Feldman BM. A wearable activity tracker intervention for promoting physical activity in adolescents with juvenile idiopathic arthritis: a pilot study. Pediatr Rheumatol Online J. 2018;16(1):66.
14. Miller GE, Chen E, Parker KJ. Psychological stress in childhood and susceptibility to the chronic diseases of aging: moving toward a model of behavioral and biological mechanisms. Psychol Bull. 2011;137(6):959-97.
15. Alley DE, Seeman TE, Ki Kim J, Karlamangla A, Hu P, Crimmins EM. Socioeconomic status and C-reactive protein levels in the US population: NHANES IV. Brain Behav Immun. 2006;20(5):498-504.
16. Lyons ER, Norman Wells J, Scholtes CM, Mintz B, Giuliano RJ, Skowron EA. Recollections of positive early caregiving relate to sympathetic nervous system activation and chronic inflammation in subsequent generations. Dev Psychobiol. 2019;61(2):261-74.
17. Danese A, Moffitt TE, Harrington H, Milne BJ, Polanczyk G, Pariante CM, et al. Adverse childhood experiences and adult risk factors for age-related disease: depression, inflammation, and clustering of metabolic risk markers. Arch Pediatr Adolesc Med. 2009;163(12):1135-43.
18. Eyberg & Funderburk. 2011 PCIT Protocol PCIT International 2011.
19. Athreya AP, Lazaridis KN. Discovery and Opportunities With Integrative Analytics Using Multiple-Omics Data. Hepatology. 2021 Aug;74(2):1081-1087. doi: 10.1002/hep.31733. Epub 2021 Jul 4. PMID: 33539039; PMCID: PMC8333231.
