# Supplementary material for: Feasibility of Digital Augmentation of Parent-Child Interaction Therapy: A Randomized Clinical Trial
Source: JAMA Netw Open. 2025 Dec 15;8(12):e2548869. doi: 10.1001/jamanetworkopen.2025.48869 (PMC12706678; doi:10.1001/jamanetworkopen.2025.48869)
Supplement: Supplement 3. — Data Sharing Statement [file jamanetwopen-e2548869-s003.pdf]

Data Sharing Statement

Romanowicz. Feasibility of Digital Augmentation of Parent-Child Interaction Therapy. *JAMA Netw Open*. Published December 15, 2025. doi:10.1001/jamanetworkopen.2025.48869

Data
Additional Information: NCT05077722
Data available: No

Additional Information
Explanation for why data not available: In this feasibility study of digital technology, the consent for study was limited as data sharing standards for wearable data from children are yet to be understood by the broader research community.
